# Hemifield-Specific Motion Extrapolation Reveals Limits of Interhemispheric Integration

**DOI:** 10.1523/ENEURO.0004-26.2026

**Published:** 2026-08-07

**Authors:** Coleman E. Olenick, Mazyar Fallah

**Affiliations:** ^1^Human Health Sciences, University of Guelph, Guelph, Ontario N1G 2W1, Canada; ^2^Canadian Action and Perception Network, Toronto, Ontario M3J 1P3, Canada

**Keywords:** motion extrapolation, motion perception, motion processing, perceptual adaptation, peripheral, sensory integration

## Abstract

The accurate perception of moving objects is a fundamental challenge for the visual system, which must compensate for neural processing delays. Motion extrapolation is a proposed mechanism whereby the brain predicts an object's future position. We investigated how an object's motion history shapes its perceived position using the flash-jump illusion in humans of either sex, in which a brief color change in a moving bar is mislocalized as further along the direction of motion. Across two experiments, we found that longer preceding motion sequences improved localization accuracy, consistent with motion adaptation. This effect occurred regardless of whether motion continued after the flash. Notably, mislocalization transiently reappeared as the object crossed the vertical midline, suggesting that motion adaptation and motion extrapolation operate independently between within each hemifield. Manipulating the length of the sequence in each hemifield in Experiment 2 confirmed that this adaptation is spatially confined to each hemifield, with limited interhemispheric transfer. The results align with a Bayesian framework in which the brain integrates signals from both hemispheres, with midline crossings triggering a shift from adapted to unadapted neural populations. We identify motion extrapolation, supported by hemifield-specific adaptation in area MT and integration in area MST, as the putative mechanism behind these midline discontinuities. This work reframes smooth pursuit not just as a tracking behavior but as a solution to overcome limitations of interhemispheric motion processing.

## Significance Statement

When we see moving objects, our brains must constantly predict where they will be next because neural processing takes time. This study shows that the prediction of motion depends on how long we observe something moving, with longer motion histories improving the predictions. However, when an object crosses from one side of our visual field to the other, these predictions briefly reset, causing a misperception of position. The findings suggest that each half of the brain adapts motion predictions separately and that they are integrated together at a later stage. These results reveal how the brain combines prediction and adaptation to keep up with a fast-moving world and the limits of these processes.

## Introduction

Accurately perceiving and interacting with moving objects is a fundamental challenge for the visual system. Inherent delays in neural processing arise from the time required for processing through the visual hierarchy, leading to misalignments between the brain's representations and real positions of objects in the world. To compensate for these delays, the visual system employs predictive mechanisms to accurately localize moving objects. For instance, when driving at high speeds, drivers must anticipate the movements of other vehicles. If neural processing were purely reactive, drivers would consistently misjudge positions; however, they regularly respond appropriately by predicting where objects will be. Motion-related perceptual illusions have been employed to study the neural compensation for objects in motion (Hogendoorn, 2020). This research led to the theory of motion extrapolation, which posits that predictions about object motion are used to shift its processing further along its trajectory across the visual hierarchy (Turner et al., 2024).

The flash-lag effect is an influential paradigm for the study of motion extrapolation (Nijhawan, 1994). In this paradigm, a bar moves across a screen at a constant speed, and a stationary bar is flashed adjacent to it; the flashed bar is perceived behind the moving bar. This paradigm has been adapted through various manipulations into flash-related motion illusions (Hogendoorn, 2020). One manipulation, the flash-jump illusion, removes the physical separation between the stationary and moving bars, instead changing the color of the moving bar. In the flash-jump illusion, the flash is perceived later than it occurred, a result dubbed the flash-jump effect (FJE; Sundberg et al., 2006; Eagleman and Sejnowski, 2007). Recent work on the FJE demonstrated that the color of the flash modulated the degree of extrapolation consistent with attentional weighting of the true flash position in a Bayesian perceptive framework (Saini et al., 2021).

Applying Bayesian principles to motion-induced position shifts is well established (Koechlin et al., 1999). Computational models have been developed with a Bayesian basis to explain the flash-lag effect (Khoei et al., 2017) and motion perception more generally (Montagnini et al., 2007; Kwon et al., 2015; Yang et al., 2021; Jiang and Rao, 2024). The central principle of Bayesian approaches to motion perception is that the brain combines noisy sensory inputs with prior expectations about the motion of an object to form a posterior estimate about that object's position (Weiss et al., 2002). Internal models of motion extrapolation are formalized as priors that are updated by recent motion and improved by coherent motion (Rao, 2004). When sensory evidence is weak, noisy, or otherwise unreliable, object localization relies more heavily on these motion extrapolation mechanisms (Stocker and Simoncelli, 2006; Khoei et al., 2017). The Bayesian perspective provides a unifying framework for understanding motion-induced position shifts like the FJE.

We are interested in how motion history affects motion extrapolation. How does the duration that an object has traveled along a given trajectory influence the localization of that object? Early work manipulating the duration of the moving bar in the flash-grab illusion, wherein a stationary object is flashed as a moving background reverses direction and is perceived to be pulled or “grabbed” in the new direction of motion. This work found conflicting results, reporting an increase (Vreven and Verghese, 2005) or decrease (Bachmann et al., 2003; Cantor and Schor, 2007) in the size of the flash-lag effect. However, Linares et al. (2007) demonstrated that the influence of duration was modulated by the distance between the moving bar and the flash. Specifically, the flash-lag effect increased with the duration of the preflash trajectory only when the flash was presented distant from the moving object; the duration had little influence when the flash was presented nearby. The spatial interactions of motion history and the position of other objects found by Linares and colleagues is part of the motivation to use the FJE, by overlapping the location of the motion and the flash. More recent work has found that the flash-grab effect is increased for longer preceding motion history (Coffey et al., 2019; Takao et al., 2022). Coffey et al. (2019) attributed the increased illusion to an increased expectation of the flash within the experimental windows. These results create a spatial separation between either the flash and the preceding motion or, in the case of Coffey et al. (2019), a spatial discontinuity between preceding and succeeding motion via the flash-grab effect. In this study, we examine the effects of motion history on motion-induced mislocalizations without spatial separation of the flash from its motion history by using the FJE. Based on prior results, we expect that longer preceding motion sequences would result in an increase in the effects of motion extrapolation producing an increase in the FJE but only when there is motion after the flash (Sundberg et al., 2006).

## Materials and Methods

Note that the methods and data from Experiment 1 have been previously published by Saini et al. (2021), but the data are being used for the novel analyses presented here.

### Participants

Twenty-four human participants volunteered for this study but were naive to the purpose (seven males; range, 18–43 years). Ethics approval was obtained from the York University Human Participants Research Committee. Written consent was acquired prior to participation, and color vision was ensured using the Ishihara test for color blindness (Ishihara, 2006).

### Procedure

Participants were sat in a dimly lit room with their head placed on a chinrest for the duration of the experiment. The chinrest was positioned 84 cm away from a 21″ CRT monitor (60 Hz, 1,024 × 768). Throughout the experiment, participants were required to maintain a fixation within a 2° width box around a cross (1° in each direction from center), which was monitored using an EyeLink II infrared eye tracker (SR Research) to track the right eye position. The experimental procedure and data collection was programmed using the Presentation software (Neurobehavioral Systems).

**Figure 1.**
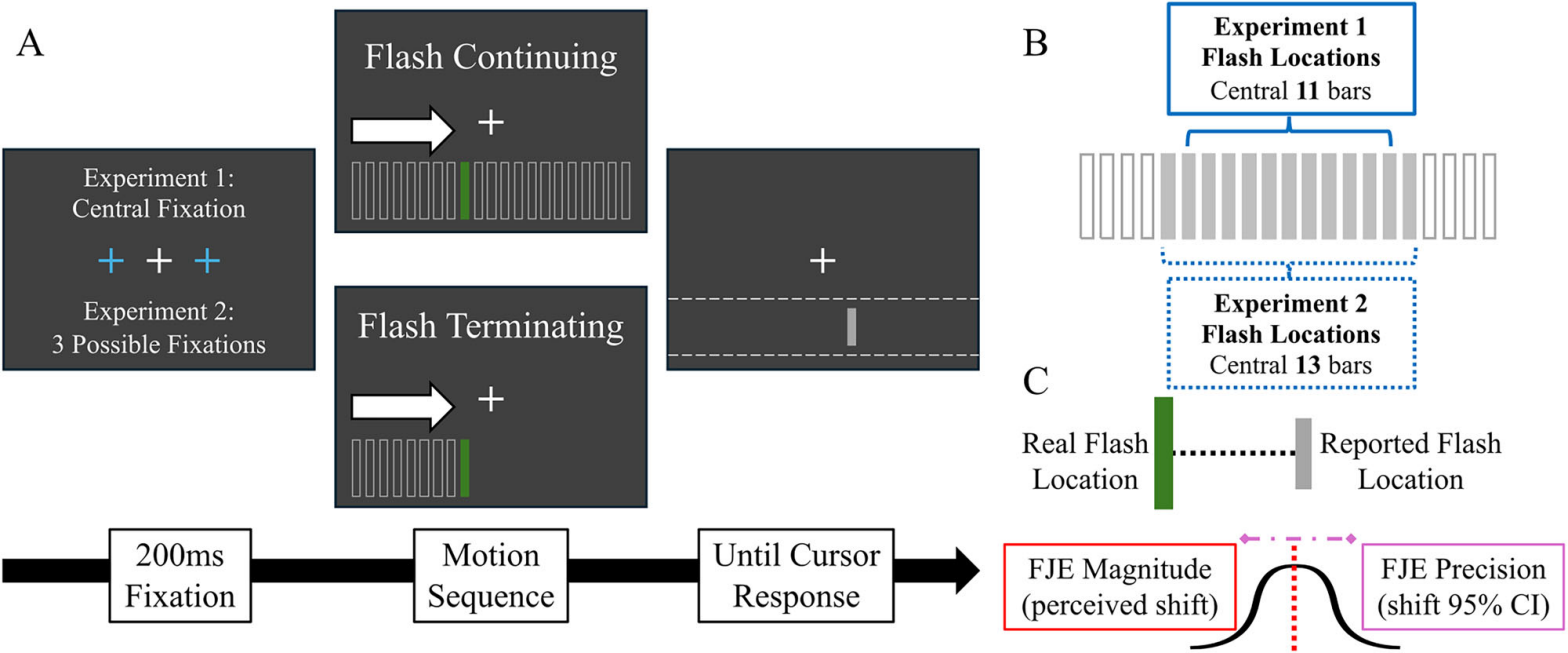
Experimental methods. ***A***, Progression of an experimental trial began with a 200 ms fixation on a cross at the center of the display (Experiment 1), or center, 4 dva to the left, or right of center (Experiment 2). Following the initial fixation period, gray bars began an apparent motion sequence from the left or right of the display (only rightwards motion depicted). One of the bars in the motion sequence was instead presented as a color (blue, red, yellow, or green in Experiment 1, only green in Experiment 2). The colored bar was either presented as the last in the sequence (flash-terminating) or with subsequent gray bars extending to the other edge of the display (flash-continuing). After the sequence ended, participants then indicated where they observed the colored flash with a vertically locked probe bar. ***B***, The colored flash could be presented at any of the central 11 bars (Experiment 1) or 13 bars (Experiment 2), with the overall length of the sequence unchanged in the flash-continuing condition. ***C***, The magnitude of the FJE was computed as the offset between the reported flash position and the actual flash position, corrected for the direction of motion (i.e., further in the direction of motion was positive while earlier was negative). A 95% confidence interval for each participant and condition was used to assess the precision of the FJE. See Extended Data Figure 1-1 for an assessment of the skew and distribution of the FJE across the flash positions.

At the start of the experiment, the eye position was calibrated and throughout as required. As illustrated in Figure 1, the trials began with the participant fixating a gray cross (0.23 × 0.23°; CIExy = [10.83, 16.25]; luminance = 11.93 cd/m^2^) presented on a black background (CIExy = [0.35, 0.56]; luminance = 0.365 cd/m^2^) for 200 ms. Following fixation, a gray bar (0.2 × 2.24°; CIExy = [10.83, 16.25]; luminance = 11.93 cd/m^2^) appeared 2.71 dva below fixation and 5.22 dva to either the left or right side of the center of the display. The bar appeared for 2 frames (33 ms) and then disappeared for 2 frames, subsequently reappearing 0.77° leftward or rightward. This sequence generates apparent motion of the bar with a speed of 11.41°/s moving left or right from the periphery over 21 bar positions[Fig eN-NWR-0004-26F2][Fig eN-NWR-0004-26F3].

10.1523/ENEURO.0004-26.2026.f1-1 Figure 1-1 **Distribution of Flash Jump Effect (FJE) Magnitude Across Flash Positions.** Violin plots depicting the distribution of FJE magnitude across flash positions for the different fixation-location and motion conditions in Experiments 1 and 2, collapsed across motion direction and shown as rightward motion. The top row shows Experiment 1 flash-continuing (red) and flash-terminating (blue) conditions. The middle and bottom row are the flash-continuing and terminating conditions from Experiment 2, respectively. Middle and bottom row panels correspond to the different fixation conditions (central [pink], early [yellow], and late [purple] fixation locations). Positive FJE values indicate perceived flash positions displaced further along the direction of motion, with larger positive values reflecting greater forward mislocalization. The width of each violin reflects the density of responses at that flash position. The horizontal dashed line at 0 indicates veridical flash localization, and the vertical dashed line indicates the flash position aligned with fixation and the vertical meridian. Download Figure 1-1, TIF file.

**Figure 2. eN-NWR-0004-26F2:**
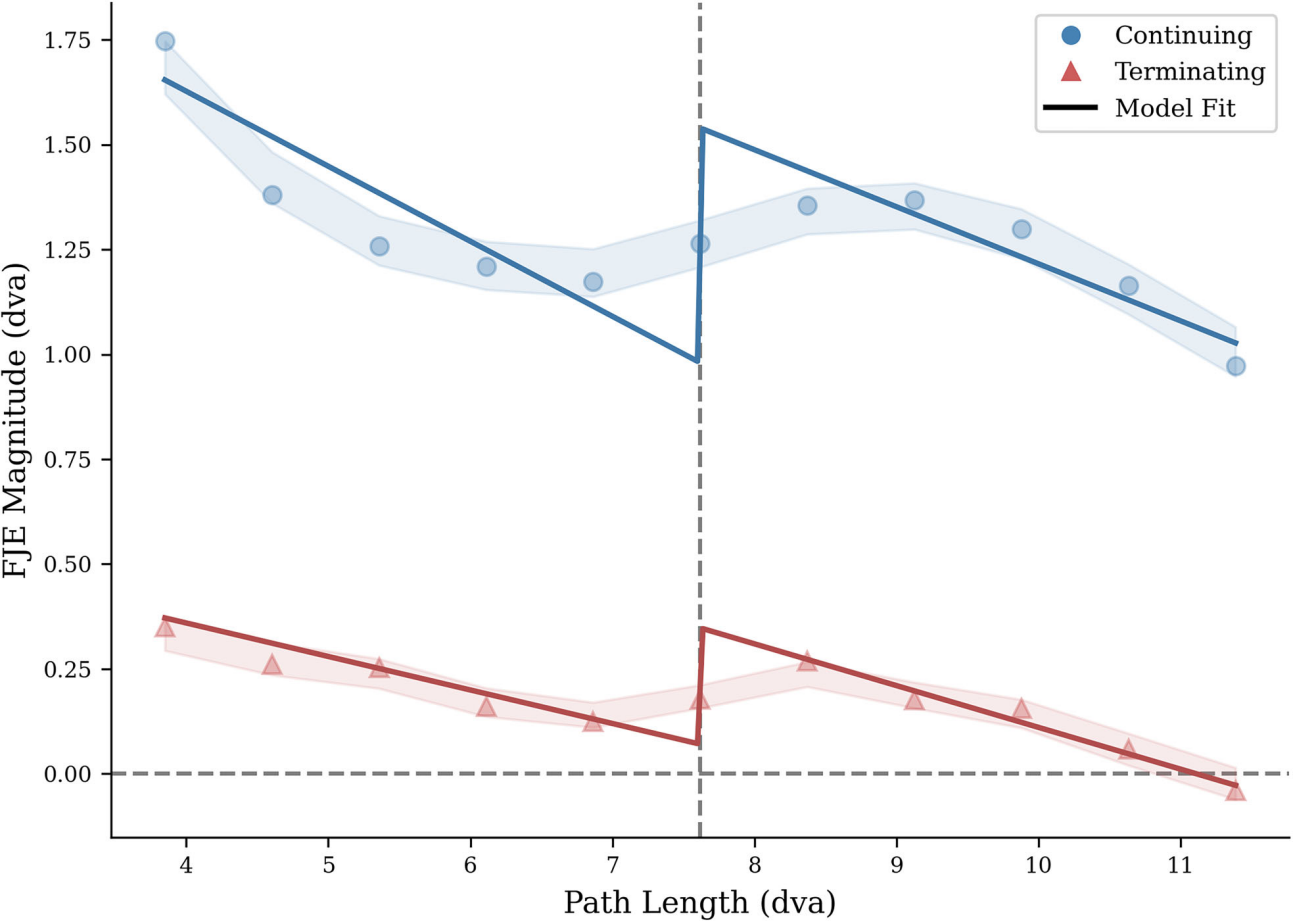
Experiment 1 FJE magnitude over flash positions. FJE magnitude plotted across the distance traveled by the bar before the colored flash, collapsed across motion direction, and depicted as rightward motion. Each point represents the mean ± standard error of the perceived shift in the flash-terminating (red triangles) and flash-continuing (blue circles) motion conditions. Positive values indicate reports of the flash position further along its motion path, with greater values reflecting less accurate perception. The horizontal dashed line at 0 represents accurate reporting of the flash position. The vertical dashed line indicates the position directly under the fixation cross and the VM. The solid lines are the predicted FJE values for the different conditions in a LME regression model across the length of the motion sequence. The model included a motion condition split (terminating and continuing) and a discontinuity at the VM. The effects of motion direction and color were assessed similarly and are reported in Extended Data Figure 2-1 and 2-2, respectively.

10.1523/ENEURO.0004-26.2026.f2-1 Figure 2-1 **Experiment 1 FJE Magnitude Linear Mixed Effects Model with Direction of Motion.** Parameter estimates and test statistics for the FJE magnitude linear mixed-effects model that includes the direction of motion. Abbreviations: Path Len, path length (dva); Term, flash-terminating condition; RW, rightward motion (other parameters use leftward motion); Post-Mid, indicator for post-midline flashes; SE, standard error; CI, confidence interval. Model fit statistics: AIC = 46820.07, log-likelihood = **-**23383.03. Likelihood-ratio comparison against a null model: χ²(24) = 5476.09, p < .001. Download Figure 2-1, DOCX file.

10.1523/ENEURO.0004-26.2026.f2-2 Figure 2-2 **Experiment 1 FJE Magnitude Linear Mixed Effects Model with Colour of Flash.** Parameter estimates and test statistics for the FJE magnitude linear mixed-effects model. Abbreviations: Path Len, path length (dva); Term, flash-terminating condition; Col-G/Y/B, colour of flash with G = green, Y = yellow, B = blue (note that a red flash was used as the base for parameter estimates); Post-Mid, indicator for post-midline flashes; SE, standard error; CI, confidence interval. Model fit statistics: AIC = 47709.98, log-likelihood = -23800.99. Likelihood-ratio comparison against a null model: χ²(51) = 4640.18, p < .001. Download Figure 2-2, DOCX file.

**Figure 3. eN-NWR-0004-26F3:**
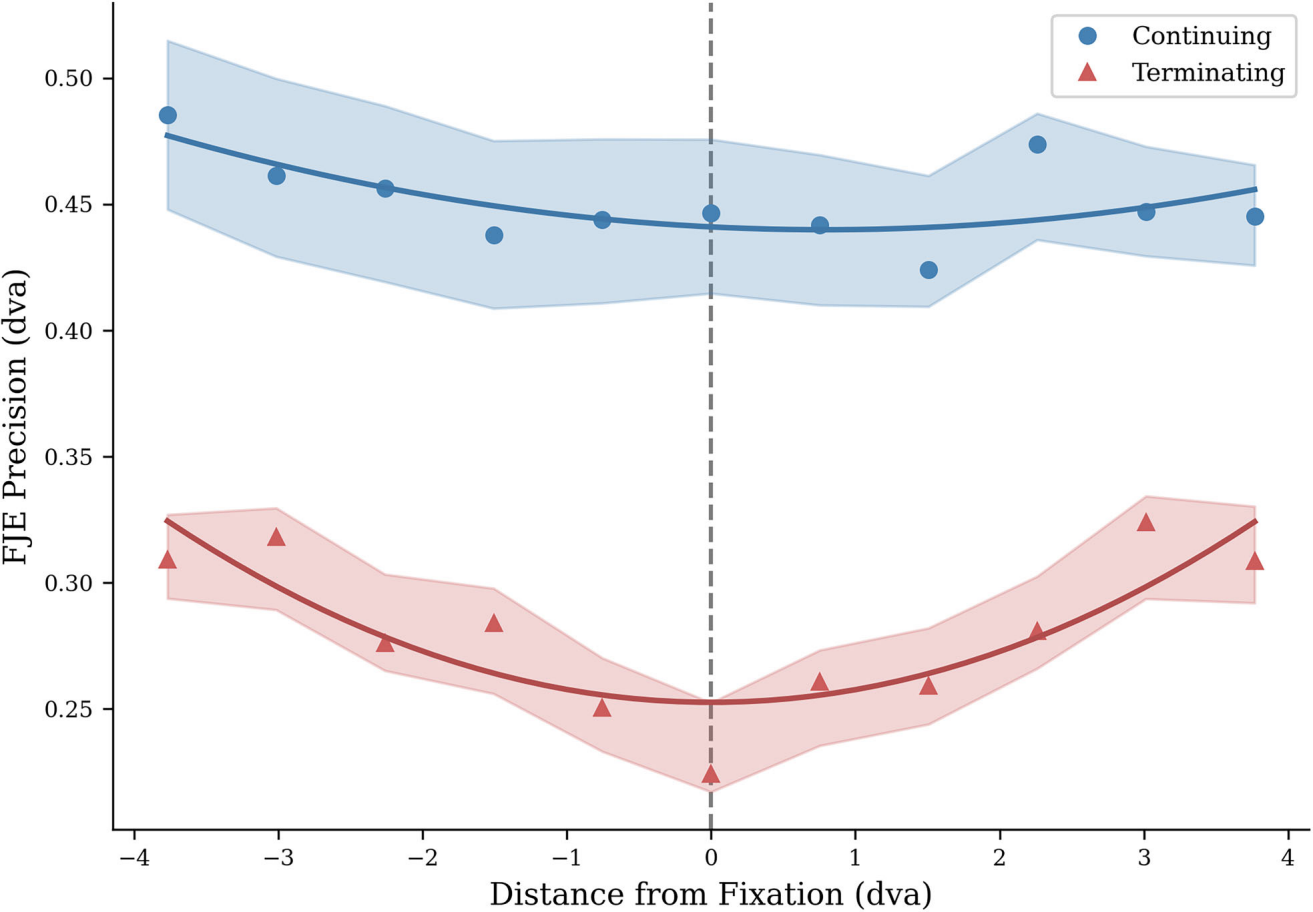
Experiment 1 FJE localization precision over flash positions. FJE precision plotted across the horizontal distance of the colored bar flash from the VM, collapsed across motion direction, and depicted as rightward motion. Each point represents the mean ± standard error of the mean range for each participant in the flash-terminating (red triangles) and flash-continuing (blue circles) motion conditions. The vertical dashed line indicates the position directly under the fixation cross and the VM. Higher values indicate greater variability in the reporting of flash position. The solid lines are the predicted FJE precision for the different conditions in an LME regression model using the eccentricity (distance from fixation cross) as a predictor. See Extended Data Figure 3-1 for a comparison of a model using the path length in place of the eccentricity.

10.1523/ENEURO.0004-26.2026.f3-1 Figure 3-1 **Experiment 1 FJE Precision Path Length Linear Mixed Effects Model.** Parameter estimates and test statistics for the FJE precision linear mixed-effects model. Abbreviations: Path Len, path length (dva); Term, flash-terminating condition; SE, standard error; CI, confidence interval. Model fit statistics: AIC = −1230.97, log-likelihood = 623.48. Likelihood-ratio comparison against a null model: χ²(3) = 49.44, p < .001. Download Figure 3-1, DOCX file.

During each trial, one of the gray bars instead appeared colored (luminance = 12 cd/m^2^; green, CIExy = [6.007, 2.516]; blue, CIExy = [27.08, 139.3]; red, CIExy = [25.57, 1.804]; yellow, CIExy = [12.24, 2.379]) at 1 of the central 11 bar positions. The motion sequence could either terminate with the appearance of the colored bar or continue after as gray bars again, yielding the terminating and continuing motion path conditions, respectively. Once the motion sequence of the bar had completed, participants were asked to indicate the position where the colored bar had appeared using a probe identical to the gray bars moved with a computer mouse. The probe was locked to the vertical position of the bars but could be moved freely horizontally, with its initial position randomized across the entire range of bar positions. During this response period, participants were permitted to move their eyes freely.

### Data analysis

To quantify the magnitude of the FJE for a given trial, the horizontal difference between the reported bar position and its actual position was calculated in degrees of visual angle, such that positive values reflect errors further along in the direction of the apparent motion of the bar. Due to substantial skew for flashes occurring near the edge of the display (Extended Data Fig. 1-1), FJEs 2.5 standard deviations from the mean within each condition were removed (416/16,896 removed [2.5%]). The FJE was collapsed across the four colors as the effects of color on the FJE have been previously reported by Saini et al. (2021). Flashes were also collapsed across motion direction as there was no interaction or main effect of the motion direction (Extended Data Fig. 2-1). The precision of the spatial localization of the illusory flash location was quantified on a participant-by-participant basis by calculating a 95% confidence interval at each experimental condition for each participant, a similar method to those used in reach and gaze paradigms (Henriques et al., 2003; Blohm and Crawford, 2007) and by Saini et al. (2021).

A linear mixed-effects (LME) regression model was used to investigate the combined effects of sequence termination conditions and flash position on the FJE magnitude and precision. An LME model allows for a comparison of variation across individual participants while accounting for general differences in magnitude of the FJE (Meteyard and Davies, 2020). The LME models were fit using the statsmodels python package (Seabold and Perktold, 2010). In line with this, random effects for main model variables were included for each participant for the magnitude models, but not for interaction terms to prevent model convergence and overparameterization errors. The FJE precision models included only random intercepts for the motion termination conditions due to the reduced quantity of data. Following a qualitative examination of the data, the overall sequence length model was refit excluding the central flash position and an additional parameter for flashes after fixation in the sequence.

The FJE precision was fit with two separate LMEs using the sequence length and eccentricity from fixation, with different intercepts for the terminating conditions. All LME comparisons were made through likelihood-ratio tests and Akaike information criterion (AIC) on maximum-likelihood fit models, with model quality overall compared with a null model (i.e., only intercepts).

### Participants

Unlike the data from Experiment 1 that has been published previously, Experiment 2 recruited a new set of 24 naive human participants (three males; 18–27) and were compensated $15 for their time. Ethics approval for the study was obtained from the University of Guelph research ethics board. Written consent was acquired prior to participation, and color vision was ensured using the Ishihara test for color blindness (Ishihara, 2006).

### Procedure

Experiment 2 followed a similar procedure with key experimental manipulations to better explore the effects of flash position (Fig. 1). Participants were seated in a dimly lit room and positioned with a display 57 cm from their eye. The position of the eye was calibrated at the start of the experiment and throughout as required. Like Experiment 1, trials began with the participant fixating a gray cross (0.4 × 0.4°; CIExy = [12.28, 18.26]; luminance = 13.49 cd/m^2^) on a black background (CIExy = [0.361, 0.5792]; luminance = 0.364 cd/m^2^) for 200 ms. They were monitored and required to stay within a 2° width box around a cross (1° in each direction from center). However, the position of the fixation cross varied between blocks and was presented at either the center of the display or 4 dva to the right or left of the center for the duration of a block.

After the required fixation, a gray bar (0.34 × 4.14°; CIExy = [12.28, 18.26]; luminance = 13.49 cd/m^2^) appeared 7.76 dva below fixation and 11.35 dva to the left or right edge of the display center (measured to the center of the bar) and followed a faster apparent motion sequence than Experiment 1 at a speed of 16.82°/s. Along the motion path of the gray bar, one of the bars instead flashed green (CIExy = [6.792, 2.781]; luminance = 13.49 cd/m^2^) at 1 of the central 13 bar positions, with the motion sequence continuing or terminating after the green bar. The broader flash and fixation positions allow for a more comprehensive examination of the effects of flash position on FJE magnitude across the visual field from 9.5 dva before to 9.5 dva after fixation in the motion path. Much like the first experiment, motion will occur in both leftward and rightward directions but, given the lack of direction effects in the first experiment, our analysis will collapse the directions. This paradigm also allows for the examination of fixations that occur at early, intermediate, and later times of the overall motion sequence (e.g., an early fixation could be left of center with rightward motion or right of center with leftward motion).

Each participant completed six blocks in total repeating each fixation cross position twice. The order of the fixation position between blocks was a randomized sequence of the three positions for each participant. Each block contained 52 trials consisting of one trial for each of the 13 flash positions, two motion directions (left vs right), and two motion path conditions (terminating vs continuing).

### Data analysis

The FJE magnitude and precision were computed in the same manner as Experiment 1 and converted into degrees of visual angle. After collapsing across motion direction, filtering of trials 2.5 standard deviations from the mean of each condition was performed (203/7,488 trials removed [2.7%]). Following from Experiment 1, an LME was fit with the path length preceding the flash, position of the fixation cross, terminating condition, and an indicator of flashes occurring after the midline as fixed effects. This model also included the interactions of these effects and random effects for each of the fixed effects, but not their interactions. The central flash positions [<1 dva from the vertical meridian (VM)] were excluded from the model fits to allow clear before and after midline crossing distinction.

Following the LMM fits, resurgence peak locations were estimated using a participant-level bootstrap smoothing procedure, separately for each fixation position and terminating condition. For each condition, raw FJE values were first averaged within participant and flash position, collapsing across repeated trials, directions, and blocks after the outlier filtering applied earlier. Bootstrap samples were then generated by resampling participants with replacement, with no additional within-participant trial resampling. On each bootstrap iteration, the resampled participant means were averaged at each flash position to produce a group-level FJE curve over the length of the sequence. There were no missing flash position values for any participant after determining the within participant means (i.e., each participant had at least one trial for each condition and flash position). The resulting curve was smoothed using a cubic Savitzky–Golay filter across a five-position window. Local peaks were identified from the smoothed curve using the “find_peaks” function from the scipy Python package with a minimum prominence of 0.05 dva to avoid small or inconsistent jumps. When multiple peaks were detected, the largest peak at or beyond the VM was prioritized; if no such peak was identified, the largest detected local peak was used. To allow for peak estimates not bound to the exact flash positions, the selected peak position was refined by fitting a quadratic function to the peak and its two neighboring points. Peak location, peak FJE, and distance from the VM were summarized as bootstrap medians with percentile-based 95% confidence intervals across 5,000 bootstrap samples. The proportion of samples in which a local peak was identified and used for the summary median and interval is reported in Extended Data Figure 5-1.

The FJE precision, quantified using individual condition and participant 95% confidence intervals, was fit using the same two models as Experiment 1: sequence length and distance from fixation (eccentricity), with the fixation position and terminating condition as fixed effects. Random intercepts and terminating condition were included.

## Results

The effects of motion history on the magnitude of the FJE were investigated using a LME regression model. The motion path (continuing vs terminating), motion sequence length (distance traveled prior to the flash), and their interaction were included as fixed effects in the LME model, with corresponding random effects per-participant for the main fixed effects (Table 1). The overall LME was a significant improvement on the null model (*χ*^2^_(8)_ = 6,022.28; *p* < 0.001), and the inclusion of random effects significantly improved the model (*χ*^2^_(5)_ = 2,880.71; *p* < 0.001). There was a significant reduction in the FJE for the flash-terminating condition (−1.1823 ± 0.1220 FJE; *p* = 3.36 × 10^−22^), as found in prior work (Saini et al., 2021). There was no main effect of path length in the continuing condition (−0.0562 ± 0.0375 FJE/dva; *p* = 0.1340), with a significantly shallower slope for the terminating condition (0.0166 ± 0.0074 FJE/dva; *p* = 0.0247).

**Table 1.** Experiment 1 FJE magnitude initial LME model

Parameter	Estimate ± SE	*z*	*p*	Random effects SE
Intercept	1.5178 ± 0.2022	7.508	6.01 × 10^−14^	0.8699
Path Len	−0.0562 ± 0.0375	−1.499	0.1340	0.1608
Path Len*Term	0.0166 ± 0.0074	2.247	0.0247	–
Term	−1.1823 ± 0.1220	−9.689	3.36 × 10^−22^	0.5088

Parameter estimates and test statistics for the FJE magnitude LME model. Abbreviations: Path Len, path length (dva); Term, flash-terminating condition; SE, standard error; CI, confidence interval. Model fit statistics, AIC = 51,130.04; log-likelihood = −25,554.02. Likelihood-ratio comparison against a null model: *χ*^2^_(8)_ = 6,022.28; *p* < 0.001.

However, we observed a discontinuity near the VM, consistent with evidence from prior motion–illusion work to suggest such a discontinuity (Liu et al., 2018; Benvenuti et al., 2020). So, we fit a similar LME that excludes the flashes occurring on the VM and with an additional indicator term and random effect, for flashes that occurred after the bar had crossed the VM (Fig. 2, Table 2). This model fit significantly better than the initial model (AIC decrease, −102.4; *χ*^2^_(8)_ = 118.35; *p* = 7.25 × 10^−22^) with the random effect of this indicator providing small but significant value as well (AIC decrease, −3.8; *χ*^2^_(4)_ = 11.83; *p* = 0.0187). When allowing for a discontinuity at the VM, there was a clear effect of path length that decreased the FJE magnitude (−0.1789 ± 0.0384 FJE/dva; *p* = 3.09 × 10^−6^) that was significantly shallower in the flash-terminating condition (0.0990 ± 0.0247 FJE/dva; *p* = 5.88 × 10^−5^). The midline discontinuity term also demonstrated a significant increase in FJE for flashes occurring after the midline (0.3980 ± 0.1200 FJE; *p* = 9.13 × 10^−4^). The decrease in FJE across path length continued with the same magnitude when crossing the midline (path length change, 0.0429 ± 0.0246 FJE/dva; *p* = 0.0815). Together, the results of this model indicate a motion adaptation mechanism that is transiently disrupted for motion across the VM, occurring regardless of whether motion continues after the flash or not. In summary, the magnitude of the FJE generally decreases as the length of the motion sequence preceding the flash increases. The reduction in FJE, together with the sudden resurgence across the midline, indicate that motion processing within a hemisphere facilitates more accurate flash localization, while hemifield switching is detrimental.

**Table 2.** Experiment 1 FJE magnitude LME model with midline indicator

Parameter	Estimate ± SE	*z*	*p*	Random effects SE
Post-Mid	0.3980 ± 0.1200	3.316	9.13 × 10^−4^	0.1963
Intercept	1.6540 ± 0.2003	8.259	1.46 × 10^−16^	0.8539
Path Len	−0.1789 ± 0.0384	−4.665	3.09 × 10^−6^	0.1476
Path Len*Post-Mid	0.0429 ± 0.0246	1.742	0.0815	–
Path Len*Term	0.0990 ± 0.0247	4.018	5.88 × 10^−5^	–
Path Len*Term*Post-Mid	−0.0626 ± 0.0348	−1.799	0.0720	–
Term	−1.2824 ± 0.1242	−10.327	5.33 × 10^−25^	0.4992
Term*Post-Mid	−0.0465 ± 0.1571	−0.296	0.7671	–

Parameter estimates and test statistics for the FJE magnitude LME model. Abbreviations: Path Len, path length (dva); Term, flash-terminating condition; Post-Mid, indicator for postmidline flashes; SE, standard error; CI, confidence interval. Model fit statistics: AIC = 46,623.25; log-likelihood = −23,292.63. Likelihood-ratio comparison against a null model: *χ*^2^_(16)_ = 5,656.91; *p* < 0.001.

Given the primary findings of Saini et al. (2021) regarding the effect of color on the magnitude of the FJE, we also investigated potential interactions of the FJE adaptation and resurgence with color. While we collapsed the direction of motion for the prior analyses, we also investigated the potential differences in the adaptation across direction and color separately (Extended Data Fig. 2-1 for direction, Extended Data Fig. 2-2 for color). Adding color to the model led to a reduction in model performance (AIC increase, +196.8; *χ*^2^_(35)_ = ∼0.0; *p* ∼ =1.0); we found an increase in the FJE for green and yellow relative to blue and red, as in Saini et al. We found no interactions of the adaptation effect with color, and only yellow showed a small significant increase in the resurgence effect, relative to the other colors (0.7432 ± 0.3212 FJE; *p* = 0.0207). Similarly, the addition of motion direction also reduced the model fit (AIC increase, +1,086.7; χ^2^_(8)_ = ∼0.0; *p* ∼ =1.0). There was a significantly stronger resurgence when crossing the midline for rightward, continuing motion (0.5157 ± 0.2234 FJE; *p* = 0.0210).

To investigate if the precision of flash localization also varies with motion history or flash position, we compared two different LMEs to the FJE precision: one with path length (Extended Data Fig. 3-1) and one with eccentricity (distance from fixation; Table 3) as fixed effects; both models also include terminating condition as a fixed effect. The eccentricity model (Fig. 3) provided a significantly better fit than the path length model (AIC decrease, −44.42). Accounting for individual effects, there was significantly lower variation in the flash-terminating condition (−0.3602 ± 0.0642 dva; *p* = 1.96 × 10^−8^), but no effect of motion sequence length (*p* = 0.253). The precision of the FJE does not appear to share the same spatial relationship that the magnitude of the illusion does nor the resurgence or midline effects.

**Table 3.** Experiment 1 FJE precision eccentricity LME model

Parameter	Estimate ± SE	*z*	*p*	Random effects SE
Ecc	0.0196 ± 0.0084	2.340	0.0193	–
Ecc*Term	0.0360 ± 0.0118	3.042	0.0024	–
Intercept	0.3477 ± 0.0509	6.835	8.23 × 10^−12^	1.9306
Term	−0.3602 ± 0.0642	−5.615	1.97 × 10^−8^	1.0170

Parameter estimates and test statistics for the FJE precision LME model. Abbreviations: Ecc, eccentricity (distance from fixation in dva); Term, flash-terminating condition; SE, standard error; CI, confidence interval. Model fit statistics: AIC = −1,275.39; log-likelihood = 645.70. Likelihood-ratio comparison against a null model: *χ*^2^_(3)_ = 93.86; *p* < 0.001.

Experiment 2 aimed to better characterize the hemispheric habituation account suggested by Experiment 1 by sampling a wider range of motion sequence lengths relative to the VM crossing. Fixation position was placed at either the left, right, or center of the screen to manipulate the length of the motion sequence preceding the VM, extending flash positions at both ends of the trajectory relative to Experiment 1 and displacing fixation 3.5 dva earlier or later along it. If the decrease in FJE with increasing path length reflects within-hemifield habituation, it should continue for flashes within a hemifield regardless of fixation position. Likewise, if the resurgence at the VM reflects a failure of habituation to transfer across hemifields, it too should remain tied to the VM crossing rather than varying with fixation placement.

**Figure 4.**
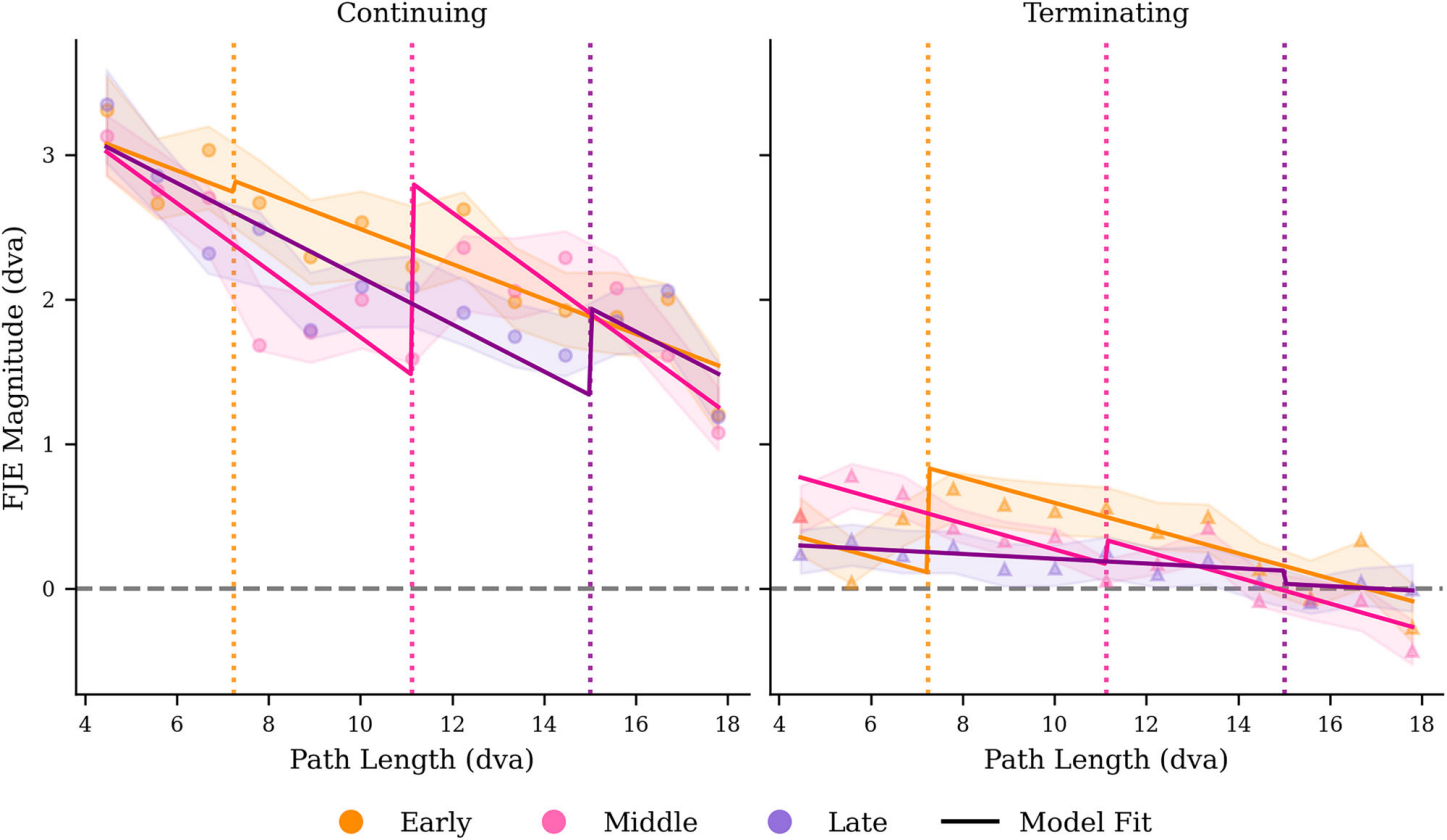
Experiment 2 FJE magnitude over flash positions. FJE magnitude plotted across the distance traveled by the bar before the colored flash, collapsed across motion direction and depicted as rightward motion. Each point represents the mean ± standard error of the perceived shift. The flash-continuing condition is shown on the left with circles and the flash-terminating condition on the right with triangles. Positive values indicate reports of the flash position further along its motion path, with greater values reflecting less accurate perception. The horizontal dashed line at 0 represents accurate reporting of the flash position. The vertical dashed lines indicate the position directly under the fixation cross and the VM. The solid lines are the predicted FJE values for the different conditions in a LME regression model across the length of the motion sequence. The model included a motion condition split (terminating and continuing) and a discontinuity at the VM. The effects of motion direction were also investigated and reported in Extended Data Figure 4-1.

We fit an LME with motion sequence length, terminating condition, fixation position, and a VM-crossing indicator as fixed effects on FJE magnitude, with all pairwise interactions and random effects for each main factor. Like Experiment 1, data within 1 dva of the midline were excluded to clearly separate pre- and postcrossing flashes. Given the absence of a significant path length and midline interaction in experiment 1, and because the distance to the midline varies across fixation conditions in Experiment 2, this interaction term was excluded to improve the interpretability of the fixation position effects. This model fit significantly better than a model without the midline discontinuity term (AIC change, −81.7; *χ*^2^_(12)_ = 105.75; *p* = 4.14 × 10^−17^) and replicated the significant improvement from the inclusion of random effects for the midline discontinuity (AIC change, −49.7; *χ*^2^_(6)_ = 61.69; *p* = 2.04 × 10^−11^). The results are reported in Table 4 with fits plotted in Fig. 4.

10.1523/ENEURO.0004-26.2026.f4-1 Figure 4-1 **Experiment 2 FJE Magnitude Linear Mixed Effects Model with Motion Direction.** Parameter estimates and test statistics for the FJE magnitude linear mixed-effects model. Abbreviations: Path Len, path length (dva); Term, flash-terminating condition; Fix Loc, fixation location within sequence (early or late; central fixation otherwise); RW, rightward motion (other parameters use leftward motion); Post-Mid, indicator for post-midline flashes; SE, standard error; CI, confidence interval. Model fit statistics: AIC = 25698.23, log-likelihood = -12772.12. Likelihood-ratio comparison against a null model: χ²(74) = 2668.04, p < .001. Download Figure 4-1, DOCX file.

**Table 4.** Experiment 2 FJE magnitude LME model

Parameter	Estimate ± SE	*z*	*p*	Random effects SE
Post-Mid	1.3271 ± 0.2581	5.141	2.73 × 10^−7^	0.1520
Post-Mid*Fix Loc (Late)	−0.7247 ± 0.3466	−2.091	0.0365	–
Post-Mid*Fix Loc (Early)	−1.2489 ± 0.3477	−3.592	3.28 × 10^−4^	–
Post-Mid*Term	−1.1612 ± 0.3561	−3.261	0.0011	–
Post-Mid*Term*Fix Loc (Late)	0.4682 ± 0.4905	0.955	0.3398	–
Post-Mid*Term*Fix Loc (Early)	1.8053 ± 0.4911	3.676	2.37 × 10^−4^	–
Fix Loc (Late)	−0.2749 ± 0.3122	−0.880	0.3786	–
Fix Loc (Early)	−0.4408 ± 0.2970	−1.484	0.1378	–
Intercept	4.0589 ± 0.3342	12.145	6.08 × 10^−34^	0.6451
Path Len	−0.2322 ± 0.0343	−6.766	1.33 × 10^−11^	0.0474
Path Len*Fix Loc (Late)	0.0690 ± 0.0369	1.871	0.0614	–
Path Len*Fix Loc (Early)	0.1111 ± 0.0366	3.035	0.0024	–
Path Len*Term	0.1421 ± 0.0413	3.441	5.81 × 10^−4^	–
Path Len*Term*Fix Loc (Late)	0.0045 ± 0.0520	0.087	0.9309	–
Path Len*Term*Fix Loc (Early)	−0.1083 ± 0.0518	−2.093	0.0363	–
Term	−2.8889 ± 0.3799	−7.604	2.86 × 10^−14^	0.5385
Term*Fix Loc (Late)	−0.5252 ± 0.4369	−1.202	0.2294	–
Term*Fix Loc (Early)	0.0133 ± 0.4074	0.033	0.9740	–

Parameter estimates and test statistics for the FJE magnitude LME model. Abbreviations: Path Len, path length (dva); Term, flash-terminating condition; Fix Loc, fixation location within sequence (early or late; central fixation otherwise); Post-Mid, indicator for postmidline flashes; SE, standard error; CI, confidence interval. Model fit statistics: AIC = 26,196.34; log-likelihood = −13,058.17. Likelihood-ratio comparison against a null model: *χ*^2^_(37)_ = 2,095.93; *p* < 0.001.

**Table 5.** Experiment 2 FJE magnitude resurgence estimates

VM-crossing position	Flash-continuing	Flash-terminating
Early	+3.98 [0.10, 8.99] dva	+1.15 [0.54, 5.07] dva
Middle	+2.52 [1.72, 4.30] dva	+1.79 [−5.50, 5.15] dva
Late	+1.23 [0.53, 1.49] dva	−4.90 [−9.69, 1.88] dva

**Figure 5.**
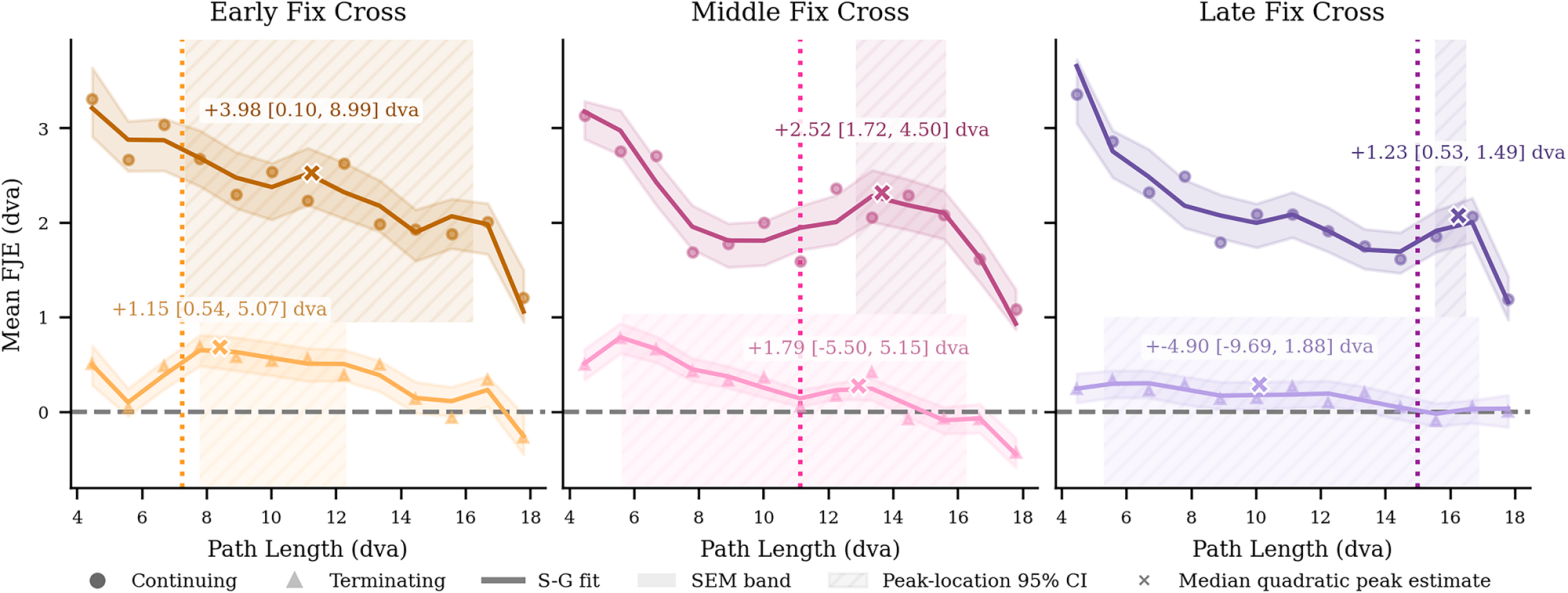
Experiment 2 FJE midline crossing resurgence estimate. FJE magnitude plotted across the distance traveled by the bar before the colored flash, collapsed across motion direction and depicted as rightward motion. The line and shaded region are the result of a Savitzky–Golay (S–G) smoothing fit with a SEM band. The flash-continuing condition (circles) is shown above the flash-terminating condition (triangles), separated by the location of the VM along the sequence: early (yellow), central (pink), and late (purple). Using a bootstrapping procedure, the point of resurgence along the path length (i.e., where the FJE increases after crossing the VM was estimated). See Extended Data Figure 5-1 for the consistency of peak identification across bootstrap samples. The 95% confidence interval for where the peak of this resurgence occurs is shown as a shaded region behind the smoothed line fit for each condition. The numeric estimates for each range are inset and reported as a median [95% CI] difference from the VM.

Peak resurgence location along the motion trajectory relative to the vertical midline (VM). Positive values indicate resurgence beyond the VM. Values are medians with 95% bootstrap CIs (5,000 resamples). See Figure 5.

We replicated the flash-terminating effect from Experiment 1, with a significant reduction in FJE magnitude in the terminating condition (−2.8889 ± 0.3799 FJE; *p* = 2.86 × 10^−14^) that did not vary with fixation position. Experiment 2 also replicated the adaptation effect, with FJE decreasing significantly with longer preceding motion path lengths (−0.2322 ± 0.0343 FJE/dva; *p* = 1.33 × 10^−11^). This decrease was attenuated for fixation positions that placed the VM crossing early (0.1111 ± 0.0366 FJE/dva; *p* = 0.0024) or late (0.0690 ± 0.0369 FJE/dva; *p* = 0.0614) in the motion sequence, relative to the central fixation. The attenuation of the adaptation effect at more extreme fixation positions may reflect the more limited range of path lengths available within each hemifield in those conditions.

There was a significant FJE resurgence when crossing the VM for the central fixation (1.3271 ± 0.2581 FJE; *p* = 2.73 × 10^−7^), which was reduced for the late VM crossing (−0.7247 ± 0.3466 FJE; *p* = 0.0365) and absent for the early VM crossing (−1.2489 ± 0.3477 FJE; *p* = 3.28 × 10^−4^) in the flash-continuing condition. The dependence of the resurgence magnitude on fixation position, rather than on a fixed screen location, supports the interpretation that the resurgence reflects a hemifield-specific process tied to the VM rather than an allocentric landmark. In the flash-terminating condition, the pattern was reversed: a resurgence was observed only when the VM crossing occurred early in the sequence (1.8053 ± 0.4911 FJE; *p* = 2.37 × 10^−4^), with no significant resurgence for central or late crossings. This interaction between fixation position and terminating condition is not straightforwardly explained by the hemispheric habituation account and warrants further investigation.

To identify where resurgences occurred and their position relative to the VM, we applied a smoothing and local peak identification procedure bootstrapped across participants (details in Materials and Methods). This confirmed the presence of VM-crossing resurgences in the conditions identified by the LME (Fig. 5). The resurgence peak location was reliably recovered for the intermediate and late VM crossings in the flash-continuing condition (Table 5), with the later fixation yielding a peak approximately half the distance from the VM as the intermediate fixation.

10.1523/ENEURO.0004-26.2026.f5-1 Figure 5-1 **Experiment 2 Bootstrap Peak Estimates Consistency Across Iterations.** The amount of iterations in which a local peak was identified for each condition of the experiment 2 bootstrap analysis procedure (see methods). The iteration counts are out of a total 5000 run for each condition. The * indicates conditions where the estimated 95% CI peak range covered no more than 3 actual flash locations. Download Figure 5-1, DOCX file.

Adding motion direction to the model significantly improved fit (Extended Data Fig. 4-1; AIC change, −498.1; *p* = 4.05 × 10^−97^). Both the adaptation and resurgence effects remained present, though varying in magnitude across directions. The early VM crossing showed a stronger adaptation effect for leftward motion, while the late VM crossing showed a stronger adaptation effect for rightward motion. In the flash-terminating condition, the adaptation effect was largely absent across directions, consistent with responses approaching a floor at accurate flash localization. Whether this reflects a true floor effect requires further examination. The intermediate VM crossing was notably consistent across directions in both adaptation and resurgence magnitude.

**Figure 6.**
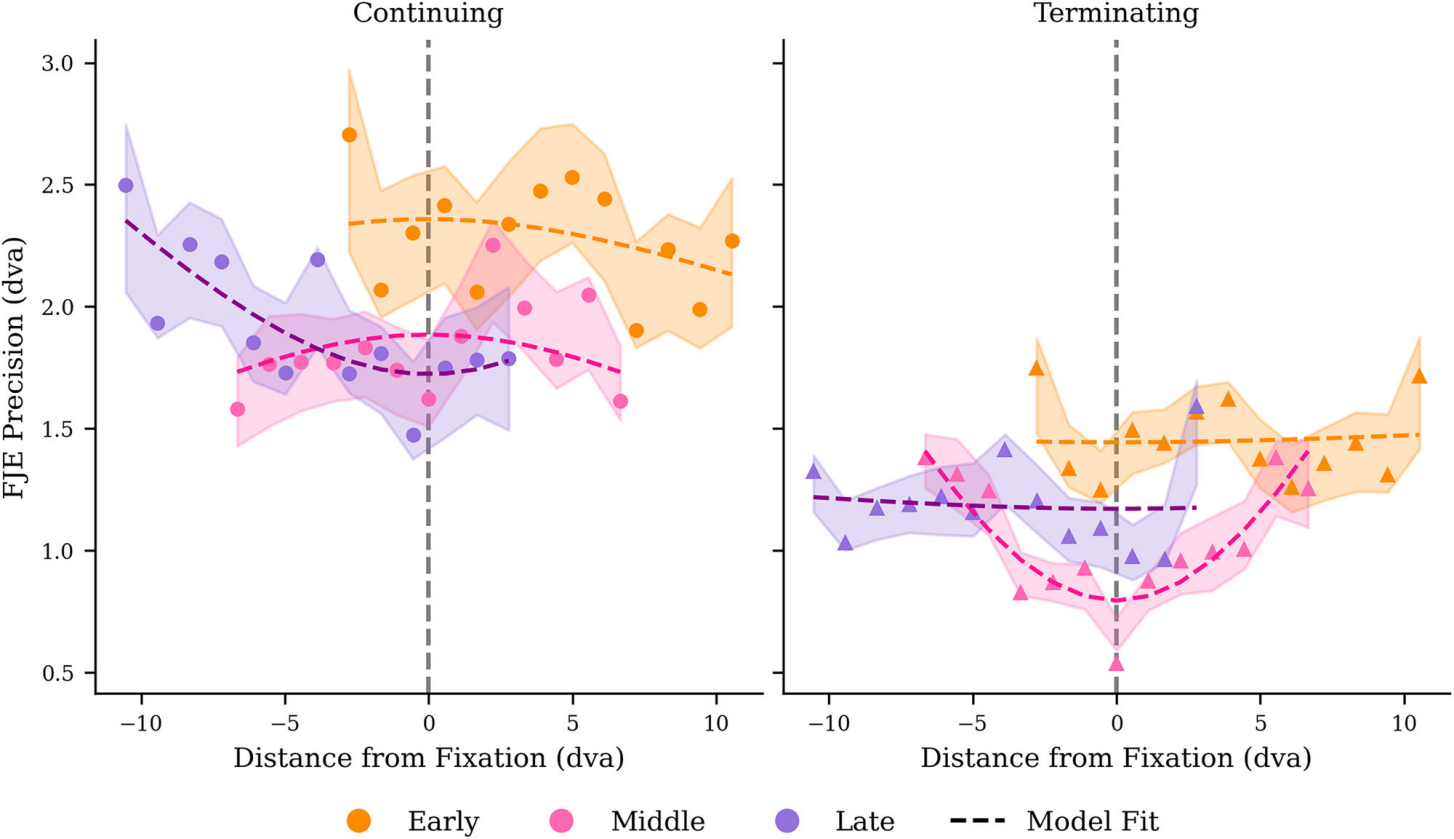
Experiment 2 FJE localization precision over flash positions. FJE precision plotted across the horizontal distance of the colored bar flash from the VM, collapsed across motion direction and depicted for rightward motion. Each point represents the mean ± standard error of the mean range for each participant in the flash-terminating (triangles) and flash-continuing (circles) motion conditions separated by the location of the VM along the sequence: early (yellow), central (pink), and late (purple). The vertical dashed line indicates the position directly under the fixation cross and the VM. Higher values indicate greater variability in the reporting of flash position. The colored dashed lines are the predicted FJE precision for the different conditions from a LME regression model using the eccentricity (distance from fixation cross) as a predictor. See Extended Data Figure 6-1 for a comparison of a model using the path length in place of the eccentricity.

The precision of the FJE was investigated using the same approach as Experiment 1, comparing an eccentricity-based LME (Table 6) to a path length-based LME (Extended Data Fig. 5-1), both including fixation position and terminating condition as fixed effects. As in Experiment 1, the eccentricity model fit significantly better than the path length model (AIC change, −21.8; Fig. 6), reinforcing the dissociation between FJE magnitude and its precision observed previously. However, the relationship between precision and eccentricity was inconsistent across fixation and terminating conditions: a significant positive relationship was found for the central fixation in the flash-terminating condition (0.3368 ± 0.084 dva/dva; *p* = 6.12 × 10^−5^) and for the late fixation in the flash-continuing condition relative to the central fixation baseline (0.1936 ± 0.066 dva/dva; *p* = 3.35 × 10^−3^). In conditions where no significant eccentricity effect was observed, precision remained near constant across flash positions. The overall inconsistency of the eccentricity–precision relationship across conditions suggests it is not a simple consequence of reduced peripheral acuity, and its origin remains an open question.

10.1523/ENEURO.0004-26.2026.f6-1 Figure 6-1 **Experiment 2 FJE Precision Path Length Linear Mixed Effects Model.** Parameter estimates and test statistics for the FJE precision linear mixed-effects model based on the length of the motion sequence. Abbreviations: Path Len, path length (dva); Term, flash-terminating condition; Fix Loc, fixation location within sequence (early or late; central fixation otherwise); SE, standard error; CI, confidence interval. Model fit statistics: AIC = 4731.15, log-likelihood = −2349.58. Likelihood-ratio comparison against a null model: χ²(11) = 144.33, p < .001. Download Figure 6-1, DOCX file.

**Table 6.** Experiment 2 FJE precision LME model

Parameter	Estimate ± SE	*z*	*p*	Random effects SE
Fix Loc (Late)	−1.8305 ± 0.6404	−2.858	0.0043	–
Fix Loc (Early)	0.2840 ± 0.6404	0.444	0.6574	–
Ecc	−0.0674 ± 0.0596	−1.132	0.2576	–
Ecc*Fix Loc (Late)	0.1936 ± 0.0660	2.934	0.0034	–
Ecc*Fix Loc (Early)	0.0220 ± 0.0660	0.333	0.7389	–
Ecc*Term	0.3368 ± 0.0840	4.009	6.11 × 10^−5^	–
Ecc*Term*Fix Loc (Late)	−0.4534 ± 0.0931	−4.868	1.13 × 10^−6^	–
Ecc*Term*Fix Loc (Early)	−0.2852 ± 0.0931	−3.063	0.0022	–
Intercept	2.4663 ± 0.5809	4.246	2.18 × 10^−5^	0.6861
Term	−3.9923 ± 0.8084	−4.939	7.87 × 10^−7^	0.5334
Term*Fix Loc (Late)	4.4453 ± 0.9042	4.916	8.83 × 10^−7^	–
Term*Fix Loc (Early)	2.6327 ± 0.9041	2.912	0.0036	–

Parameter estimates and test statistics for the FJE precision LME model. Abbreviations: Ecc, eccentricity (distance from fixation in dva); Term, flash-terminating condition; Fix Loc, fixation location within sequence (early or late; central fixation otherwise); SE, standard error; CI, confidence interval. Model fit statistics: AIC = 4,709.41; log-likelihood = −2,338.71. Likelihood-ratio comparison against a null model: *χ*^2^_(11)_ = 166.07; *p* < 0.001.

## Discussion

Across two experiments, we used the flash-jump illusion to show that longer preceding motion sequences before a flashed featural change resulted in reduced forward mislocalization and more accurate perception of the flash location. We attribute this effect at the behavioral level to adaptation to the consistent motion of the bar leading to decreased motion extrapolation. The motion adaptation effect occurred regardless of whether motion after the flash continued, though it was more prominent in the flash-continuing condition. Note that we refer here to behavioral motion adaptation (Snowden, 1998) rather than neuronal adaptation responses (Kohn and Movshon, 2003) directly. These findings are consistent with a Bayesian account in which the FJE reflects integration of an accurate position estimate with an extrapolation motion-based signal (Sundberg et al., 2006; Khoei et al., 2017; Saini et al., 2021), where adaptation reduces the contribution of the motion signal over time. In Experiment 2, by moving the fixation position while maintaining constant head position, we gathered additional supporting evidence that this adaptation occurs locally within a hemifield with limited transfer to the opposite hemisphere. This is consistent with evidence that regions near the VM are represented in both hemispheres (Fendrich et al., 1996). This produced a transient resurgence of the FJE as the bar crossed the VM. Notably, the resurgence was only apparent for intermediate and later crossing conditions, suggesting that some degree of adaptation must occur before a resurgence can be observed. However, the size of the resurgence was largest for the intermediate sequence length, indicating that some transfer of adaptation can reduce the temporary increase in mislocalization.

While our adaptation interpretation conflicts with the conclusions of Coffey et al. (2019), results from both studies indicate a stronger effect of motion history for short motion durations (<1 s) that are analogous to work in the flash-lag effect (Cantor and Schor, 2007). Coffey et al. propose that it is the expectation of the target appearance in their study rather than adaptation that leads to the increase in the magnitude of the flash-lag effect. However, the expectation of motion reversal may cause a buildup of an internal representation of the reversal. This would result in competing motion signals, where the reversal has not undergone any adaptation, and the mislocalization is due to the imbalanced combination of these signals. In our paradigm, the motion signal is constant for the object which may be why we observe a consistent decrease in magnitude across the entire sequence. Furthermore, the resurgence at the midline is not a prediction of an expectation-based account, making adaptation a more plausible explanation than expectation for our results. However, we do not test the extended motion durations of Coffey and colleagues or shifted flash windows, making it difficult to make a definitive claim or differentiation between adaptation and expectation.

There have been a multitude of theories of motion perception that we consider here in the context of our two key findings: (1) that preflash motion history reduces the FJE through adaptation and (2) that the FJE transiently resurges as the bar crosses the VM. Because the flash-jump paradigm involves a featural change within a single moving object, theories invoking attentional mechanisms triggered by the appearance of a second object, like the attentional shifting hypothesis (Baldo and Klein, 1995), cannot account for the FJE.

The differential latency hypothesis (Whitney and Murakami, 1998) proposes that moving stimuli are processed with shorter neural latency than flashed or static stimuli. Neural adaptation to the consistent motion of the bar would reduce the effective response in motion-selective areas, possibly reducing the latency advantage of the moving bar and producing the decrease in FJE magnitude we observe. Our adaptation finding can in principle be explained through differential latencies. However, the midline resurgence is harder to accommodate as there is no natural mechanism by which hemifield-specific adaptation would produce a sudden increase in the latency advantage of the bar at the VM, particularly one that scales with the length of the premidline motion sequence.

The discrete sampling hypothesis (Schneider, 2018) posits that position estimates are taken from the latest point within discrete time windows. Shorter windows with longer motion history could produce more accurate localization, and it could be further argued that separate window sizes are maintained within each hemifield yielding two independent position estimates. The combination of these estimates would produce the reported flash positions, a structure that shares a conceptual parallel with hemispheric signal integration accounts of the resurgence. However, this would predict a sharp step change at the meridian rather than the smoother resurgence we observe, which better reflects a transition between adapted and unadapted signals.

The postdiction account (Brenner and Smeets, 2000; Eagleman and Sejnowski, 2000, 2007) supposes that post-flash information biases localization along the direction of motion. Granting that temporal integration can operate over substantially longer intervals than originally proposed (Herzog et al., 2020), a postdiction account could suppose that the inclusion of a shorter sequence after the flash, as a result of a longer prior sequence, could result in less forward bias. However, this would not explain the adaptation being observed in the flash-terminating condition as well, as there are no bars presented after the flash, making a postdiction-based explanation implausible for our results.

Thus, while several theories can accommodate general adaptation effects, none readily explain the combination of preflash motion history modulation and hemifield-specific resurgence at the VM. The motion extrapolation account of motion-induced position shifts (Nijhawan, 1994; Hogendoorn, 2020), formalized through Bayesian and Kalman filter frameworks (Kwon et al., 2015; Khoei et al., 2017), provides the most coherent explanation for our main findings and discuss the implications of our results in both neuroanatomical and computational contexts. We first outline the computational basis of this account before turning to the neuroanatomical constraints that motivate our interpretation of the hemifield-specific adaptation and midline resurgence.

Motion extrapolation has been modeled previously through Bayesian inference where the motion history of an object is used as a prior to determine the actual position of the flashed object (Kwon et al., 2015; Khoei et al., 2017), a framework previously applied to the FJE by Saini et al. (2021). As proposed by Kwon et al. (2015), this can be formalized as a Kalman filter in which noisy motion priors are integrated with a position prior. In the context of our results, longer motion histories reduce the noise associated with the motion prior, allowing for better retroactive estimation of the position of the flash. That is, reductions in the prior noise result in more accurate localization, observed as the adaptation. In the flash-terminating condition, there is still a reduction in noise, but its effect is less dramatic due to a reduction in the overall weight associated with the motion prior and a greater reliance on the relatively accurate position estimate from V4 (Sundberg et al., 2006). The observed resurgence at the VM can be explained by a sudden increase in the noise of the motion prior when the bar crosses hemispheres. We propose that the motion prior in this model reflects the average of two hemispheric priors: one from the hemisphere that has processed the prior motion path and has habituated and one from the hemisphere newly exposed to the motion of the bar. The weight of each prior is determined by the reliability of the corresponding MT signal, such that greater adaptation leads to lower firing rates and, inversely, higher weight for that hemisphere's extrapolation signal. The shortest motion sequences do not have substantive adaptation yet and thus no resurgence, while the longest have only a small resurgence because of the higher weight associated with the adapted hemisphere's prior. An important alternative is that there is slow transfer of adaptation between hemispheres before crossing occurs; thus the longest sequences would have both time for adaptation to occur and for it to “leak” to the other hemisphere. This interpretation is consistent overall with the basic Bayesian principle that predictions are weighted by signal reliability (Kwon et al., 2015).

We propose that the motion adaptation observed in our study is due to neural habituation affecting the magnitude of the motion extrapolation signal in motion-selective area MT. This is supported by the magnitude of the flash-lag effect being increased or decreased through TMS (Maus et al., 2013a,b) and tDCS (Wang et al., 2022) over MT+. Additionally, the increase in the strength of both adaptation and the size of the resurgence for rightward motion (extrapolation from right to left hemisphere) supports some lateralization of motion extrapolation and thus the interhemispheric adaptation. This has been proposed recently by Suzuki et al. (2023) using the flash-lag illusion. The transient disruption of the adaptation when crossing the VM with a continued decrease after suggests that there is a transition from a more to a less adapted motion signal. However, the lack of a complete reset in the FJE to starting magnitude and bars flashed before the midline being perceived as though they had crossed (see Fig. 5 for FJE that is greater than the distance to the midline) suggest that there is some integration of multiple motion signals across hemispheres.

Given that the adaptation we observed was limited within a hemifield, it is unlikely that MST is the source of the adaptation due to that area's large bilateral receptive fields (Raiguel et al., 1997; Huk et al., 2002). In contrast, the receptive fields in MT show only limited representation of the ipsilateral visual field (Raiguel et al., 1995), consistent with our results. Furthermore, adaptation of MT has been shown to reduce perceived speed (Krekelberg et al., 2006), which suggests that speed processing being reduced through adaptation may be the driver for the reduction in motion extrapolation.

The question remains: where in the visual hierarchy are the color-change and motion extrapolation signals integrated? Motion extrapolation signals are present and selective in MT, while the representation of the colored flash arises in V4, both of which have strong hemifield separation. Given the known connections between V4 and MT (Maunsell and van Essen, 1983; Ungerleider et al., 2008), the most parsimonious site for the initial binding of the flash and moving bar is MT itself. However, this integration alone could not adequately explain our finding of the resurgence in the adaptation effect. Instead of observing a consistent magnitude for the resurgence, as would be expected from a clear hemispheric separation, we observed a smoother transition between one adaptation signal to another. As discussed in the computational account above, the combination of two adapting motion extrapolation signals, integrated downstream of the initial binding in MT, provides a more plausible explanation.

It is important to note, however, that motion extrapolation is not entirely under the purview of MT, with anticipatory responses arising at multiple stages of the visual hierarchy through feedforward and lateral connectivity as early as V1 (Jancke et al., 2004; Benvenuti et al., 2020). Liu et al. (2018) interpreted disruptions to motion-induced position shifts at both meridians as reflecting lateral connectivity within retinotopic areas, consistent with contributions from early visual areas. However, the flash-jump position shift recorded in V4 by Sundberg et al. (2006) is much smaller than the mislocalization reported by observers, indicating that ventral stream and early area connectivity alone are insufficient to account for the full magnitude of the FJE. Because the FJE requires binding a featural change to a motion trajectory, the perceptual integration must involve areas capable of representing both signals together. The resurgence we observed at the VM is more consistent with an interhemispheric boundary at the level of MT than with the meridian discontinuities of early retinotopic areas such as V2 and V3.

We therefore propose that MST is a plausible site of integration for the observed resurgence, acting as a downstream integrator of the adapting signals from MT populations in both hemispheres. MST receives inputs from MT and consists of neurons with large, bilateral receptive fields (Raiguel et al., 1997; Amano et al., 2009). In our explanation, the MST integration of motion signals from MT assigns weights to them based on their reliability to create a persistent representation of the motion. The adaptation in MT thus serves as an indicator of reliability, whereby more adapted signals correspond to more reliable motion estimates, consistent with the Bayesian framework outlined above (Kwon et al., 2015). We emphasize that this is a hypothesis consistent with the known anatomy and receptive field properties of MST, not a localization claim; our data do not directly identify MST as the integration site, and alternative loci involving feedback from higher areas or interhemispheric connectivity within MT itself cannot be excluded.

One possibility for the resurgence that does not invoke motion extrapolation or similar mechanisms is a switch from foveofugal to foveopetal motion which has been shown to modulate the flash-lag effect (Kanai et al., 2004). However, given that the stimulus motion moves primarily tangential to fixation (i.e., neither directly foveofugal or foveopetal), we do not believe that this transition would result in such an increase in the FJE. The findings of Kanai et al. (2004) would also indicate that the size of the illusion should decrease when transitioning from foveopetal to foveofugal motion, while we observed the opposite. Lastly, their study used flashed bars outside the eccentricity range that we observed the resurgence (>5 dva from the VM).

Our findings also have implications for perceptual estimates of speed and object tracking. The accuracy of tracking an object that crosses between hemifields is decreased relative to those that stay within a hemifield (Alvarez and Cavanagh, 2005), a cost attributed to early sensory processing (Minami et al., 2019). Our results indicate that the within-hemifield tracking advantage is likely due in part to separate adaptation to the moving objects in each hemisphere. This perceptual limitation may serve as a driver of an important oculomotor behavior: smooth pursuit eye movements. By pursuing a moving object, an observer stabilizes its position on the fovea, ensuring that it is represented continuously by both hemispheres (Nijhawan, 2001). We predict that even partial smooth pursuit would prevent the abrupt crossover between the adapted and unadapted hemisphere and lead to a reduced or absent resurgence when pursuit of the motion stimulus occurs.

This distinction may reflect the different goals of “vision for perception” and “vision for action” (Goodale and Milner, 1992). Our fixation-based task probes only the perceptual system, highlighting the circuitry via the limitations like the perceptual cost of crossing the midline. This framework is supported by recent findings demonstrating that different motor actions respond different to motion illusions. Specifically, work from Lisi and Cavanagh (2024) found that interceptive saccades are less influenced by illusory motion than smooth pursuit or fixated perceptual reports. However, they also found that smooth pursuit eye movements more closely resembled the biases found in fixated perceptual reports, suggesting that perception and pursuit share common motion representations (Lisi and Cavanagh, 2024). This dissociation offers a more nuanced view of the perception-action model. Rather than a strict dichotomy, visual processing may operate on a continuum of behavioral goals, supported by the hierarchy of neural timescales (Murray et al., 2014; Lisi and Cavanagh, 2024). On one end, rapid interceptive saccades represent purely action-based perception, relying on motion signals extrapolated only over short intervals (Lisi and Cavanagh, 2024). On the other end, fixated perception may integrate longer timescales to provide more stable, detailed representations. Smooth pursuit falls somewhere between, making use of longer integration windows like fixated, yet serves the action-oriented goal of stabilizing a moving stimulus. We propose that the MST, given its representation of self-motion absent in MT (Inaba et al., 2007) and its large bilateral receptive fields, is well positioned to integrate hemispheric motion signals by selectively weighting inputs based on the temporal integration required for the current goal.

Finally, while motion extrapolation provides the most parsimonious account of the FJE as presented here, cross-illusion generalizations should be made with caution. Recent work has found limited behavioral and neural correlations across motion-position illusions within individuals (Cottier et al., 2023, 2025). This suggests that paradigm similarity does not necessarily imply shared mechanisms. The absence of correlation with a variant of the FJE in that work is worth noting, though the methodological differences, simultaneous bars with size changes rather than a featural change within a single moving object, make direct comparison difficult.

By examining the FJE across various motion paths, we have demonstrated that motion adaptation and motion extrapolation occur independently in each hemifield, influencing the perception of an object's position. We argue that this phenomenon is best explained by a motion extrapolation mechanism that habituates within the hemispherically separated areas of MT. The resurgence of the illusion at the visual midline reflects a Bayesian integration of signals from both the adapted and unadapted hemispheres, a process likely occurring in area MST. These findings, observed during fixation, reveal a fundamental constraint of the “vision for perception” system with moving stimuli. More importantly, they highlight the functional significance of the “vision for action” system, suggesting that the drive to smoothly track an object with our eyes is a behavioral solution to overcome perceptual discontinuities near the midline. Our work supports motion extrapolation as the mechanism underlying these motion-induced illusions and reframes the cost of interhemispheric transfer as a driver of oculomotor behavioral strategies.

## Synthesis

Reviewing Editor: Niko Busch, Westfalische Wilhelms-Universitat Munster Decisions are customarily a result of the Reviewing Editor and the peer reviewers coming together and discussing their recommendations until a consensus is reached. When revisions are invited, a fact-based synthesis statement explaining their decision and outlining what is needed to prepare a revision will be listed below. The following reviewer(s) agreed to reveal their identity: Matteo Lisi, Alex Holcombe.

# Synthesis

Your manuscript has now been evaluated by two expert reviewers. Both find the empirical observations interesting and potentially informative: the decrease in flash-jump effect (FJE) magnitude with longer motion sequences, and especially the transient resurgence of the FJE as the moving bar crosses the vertical meridian, are acknowledged as intriguing findings. However, both reviewers also raise substantial concerns, and I agree that these must be addressed carefully before the manuscript can advance.

The most pressing issue raised by both reviewers is the potential confound between eccentricity and the adaptation account. Both reviewers also ask the authors to reconsider the theoretical framing. Moreover, a number of statistical and methodological concerns require attention. Please revise the manuscript accordingly and provide a detailed point-by-point response to the reviewers' comments.

# Reviewer 1

This manuscript reports two experiments using the flash-jump illusion to investigate how apparent motion influences position judgements. In the flash-jump illusion, a moving bar briefly changes colour, and observers typically report the colour change as occurring ahead of its true position along the direction of motion. The authors use their two datasets to argue that (i) the precision of position judgements increases as a function of the duration of motion preceding the flash (colour change), and (ii) when the bar crosses the vertical meridian, the illusion transiently re-emerges ("resurgence"). They interpret this pattern as evidence for within-hemifield motion adaptation mechanisms with limited interhemispheric transfer.

I find the question interesting and the data potentially informative, but I see several issues and possible confounds that I think the authors should address more directly.

1. Eccentricity and midline as potential confounds

A major concern is that the midline positions are also closer to the fovea / central vision due to the layout of the display. As a result, several of the key findings-greater precision and smaller mislocalisations around the midline-are exactly what one would expect from basic eccentricity effects, independently of any hemifield-specific adaptation.

Relatedly, the vertical meridian may itself act as a spatial landmark, which could improve localisation or bias reports around that region. This is particularly relevant for the first main claim (increased precision and reduced bias closer to the midline). As it stands, I am not convinced that the analyses and design fully disentangle:

- distance from fixation (retinal eccentricity),

- distance from the vertical meridian / hemifield boundary, and

- "sequence length" as an adaptation variable.

Experiment 2 may help, but I think the authors should explicitly compare modelling of precision as a function of e.g. 'eccentricity relative to fixation' versus 'sequence length'

This confound does not fully explain the resurgence effect across the midline, but it does weaken the interpretation of the basic precision and bias gradients as evidence for adaptation per se.

2. Relation to previous work on meridian crossings

There is prior work that has already examined how motion-induced position shifts behave at meridians. In particular:

Liu, S., Tse, P. U., &Cavanagh, P. (2018). Meridian interference reveals neural locus of motion-induced position shifts. Journal of neurophysiology, 119(6), 2091-2099. https://doi.org/10.1152/jn.00876.2017

Liu et al. reported that position extrapolation for moving targets does not readily cross horizontal and vertical meridians, and interpreted this in terms of lateral connectivity in retinotopic maps. This seems relevant to the present paper's claims about hemifield-specific adaptation and the neural locus of the effect

3. Bayesian / predictive frameworks: missing key reference

The discussion of Bayesian models of motion and position does not mention this key reference:

Kwon, O. S., Tadin, D., &Knill, D. C. (2015). Unifying account of visual motion and position perception.PNAS, 112(26), 8142-8147. https://doi.org/10.1073/pnas.1500361112

Kwon et al. propose that motion-induced position shifts arise from an object-tracking mechanism that can be formalised as a Kalman filter. Importantly, in a Kalman filter, the full history of observations is combined with a motion model such that position estimates become progressively less uncertain over time.

This seems directly relevant here: a Kalman-filter-like model could naturally explain the observed increase in precision with longer motion histories (even if it does not by itself explain the resurgence across the midline). It would be useful if the authors explicitly connect their findings to this framework.

4. Statistical analyses

Several aspects of the analysis would benefit from clarification and some revision.

4a. Use of medians with mixed-effects models

The authors write that "the median for every participant was calculated in each of the flash positions and motion sequence termination conditions," and then they fit linear mixed-effects models. Collapsing to condition-wise medians discards trial-level variability and largely defeats one of the main advantages of mixed-effects models: using all the data without prior averaging.

Unless there is a strong reason to do otherwise, I would recommend re-running the mixed-effects analyses on the trial-wise data (with appropriate distributional assumptions / link functions if needed). This would be both more statistically efficient and more standard practice for multilevel modelling.

4b. Coding of predictors

The regression tables are hard to interpret because it is not always clear how predictors were coded; e.g was fixation position simply the horizontal coordinates of fixation? Is sequence length expressed in number of steps, seconds, or space on the screen?

For clarity, the authors should explicitly describes the coding of each predictor and interaction in the models.

4c. Random intercepts only

It appears that only random intercepts were included. Not modelling random slopes for key within-participant factors (sequence length, condition) risks anti-conservative inference (see Barr et al., 2013). I believe the analyses should be run with random slopes; at the very least, the authors should justify why random slopes were not included, and/or report model comparisons showing that including maximal or near-maximal random effects structures does not qualitatively change the conclusions.

Barr, D. J., Levy, R., Scheepers, C., &Tily, H. J. (2013). Random effects structure for confirmatory hypothesis testing: Keep it maximal. Journal of Memory and Language, 68(3), 255-278. https://doi.org/10.1016/j.jml.2012.11.001

# Reviewer 2

Summary &Overall Assessment

This manuscript investigates how motion history shapes perceived position in the flash jump effect (FJE) using two experiments (at least one of which was published before, with this being a re-analysis, but it should be made clear whether E2 is completely new data). The core empirical contributions are: (i) a robust decrease in the flash jump effect (FJE) with longer pre flash motion (consistent with motion adaptation) and (ii) a resurgence of the FJE as the moving stimulus crosses the vertical meridian, interpreted as evidence that adaptation/extrapolation are hemifield specific The analyses primarily use LME models; for Experiment 2 the pre midline decay is modeled exponentially, and precision (95% CI width) is modeled with a quadratic term centered on fixation.

The authors acknowledge a foveopetal/foveofugal confound in the discussion but do not argue against it effectively. This confound appears to be a live candidate explanation of their results and therefore should be mentioned in the paper abstract.

Overall, the data are valuable, but several issues need attention before the claims are fully supported, both various specific issues detailed below, the foveopetal/fugal confound, and overall framing and interpretation.

Major Comments

1) Theoretical framing: avoid over generalizing extrapolation across distinct illusions

• The Introduction leans heavily on motion extrapolation as a unifying account (FLE, FJI/FJE, flash grab), culminating in strong language in the Discussion ("solidifies motion extrapolation as the key mechanism underlying these motion induced illusions"). This is too strong given ongoing debates about the FLE and related phenomena and dissociations between them (e.g. Linares &Holcombe, 2008). Please tone down and qualify these claims (e.g., "is consistent with" / "supports a role for"). For example, the last paragraph of the Introduction is not clear on the difference between them, as if they are all the same.

• Suggest acknowledging that cross illusion correlations are weak at the individual level, arguing against a single common mechanism (e.g., FLE vs flash grab vs flash jump). The recent evidence in Journal of Vision (2023) and EJN (2025) finds little to no correlation in illusion magnitudes across participants, recommending caution when generalizing mechanisms across illusions. (Provide these citations to the authors: Cottier et al., 2023; Cottier et al., 2025.)

• Relatedly, note prior arguments that the FLE is unlikely to be caused solely by extrapolation (e.g., Holcombe &Corbett https://osf.io/preprints/psyarxiv/swzr7), and that multiple mechanisms (e.g., binding, attention, positional uncertainty) may contribute depending on the paradigm.

• A paragraph in the Discussion on page 18 briefly mentions several other theories of motion-position phenomena and dismisses them on the grounds that none of them predict the sort of results found here. However, adaptation would be acknowledged by almost all theories even if it wasn't explicitly discussed, so we think this paragraph is glib. And the last part of the paragraph rules out postdiction partly on the basis of the 60-80ms window initially proposed for it, but much subsequent work, e.g. by Herzog with "shine through" Vernier displays finds evidence for a much longer window.

• the last paragraph of the Introduction mentions Linares et al. (2007) finding that for flash-lag, there was no effect of duration when the flash was presented nearby, but this is never returned to when the authors give their overall theory . And it may have even motivated the authors' choice of studying the FJE, although that , the reason for studying the FJE specifically, is also not made clear.

• In addition, like most of the literature this discussion dismisses theories too quickly because some can't explain ALL results in the literature, but that doesn't mean that multiple theories' proposed processes don't exist, they each may be making a separate contribution, as argued by Holcombe &Corbett in a preprint (https://osf.io/preprints/psyarxiv/swzr7).

Action: Temper global claims; add a paragraph in the Introduction acknowledging the multi mechanism landscape and cross illusion dissociations (cite Cottier et al., 2023/2025; potentially Holcombe &Corbett preprint).

2) An alternative interpretation of the resurgence is that it is not entirely due to hemisphere-specific adaptation, but also results simply from poor inter-hemispheric communication which impairs extrapolation (although as we mentioned in point 1, there is reason to think extrapolation is not operating). The findings of Giancini et al., 2025 preprint, which are interpreted in this way, hasn't been brought in anywhere. They studied the FLE and also found a resurgence after crossing the vertical meridian, and would be a nice addition to support their extrapolation arguments; i.e., horizontal activation in earlier areas before the integration of motion and colour change up in MT, MST etc.

3) The reduction in effect with sequence length seems to be contrary to what Bayesian accounts would predict. With a longer and longer sequence, one would think that a Bayesian estimate of the velocity and position would get stronger , have higher confidence (prior probability), and thus if it is oriented toward extrapolation, the extrapolation would increase, not decrease. More generally, we think the authors' invoking Bayesian account is not adequately justified, and the paper should arguably be written with few mentions of Bayesian.

4) Priority citation for the flash jump effect

Please cite Cai &Schlag (2001) when introducing the flash jump effect; this VSS abstract is the earliest report and is widely credited with the discovery. (Your current sentence cites Sundberg et al., 2006; Eagleman &Sejnowski, 2007.)

5) Meridian terminology and an internal inconsistency about resurgence strength

• There is confusion between vertical and horizontal meridian. The phenomena you study-hemifield crossings and interhemispheric integration-are about the vertical meridian. Yet the text/legends sometimes say "horizontal meridian" (e.g., Fig. 2 legend; Fig. 25 legends). Please standardize to vertical meridian throughout unless a specific analysis truly involves the horizontal.

• Contradictory statements about resurgence magnitude: In Experiment 2, you write that the resurgence is "increasing ... the earlier the midline was crossed" (i.e., shorter pre midline path → stronger resurgence). A few lines later you write "Thus, the more habituation that occurs before crossing the midline leads to more recovery ... (i.e., longer pre midline path → stronger resurgence). These statements conflict. Please reconcile and ensure figures/statistics match the final claim.

6) Modeling choices: central tendency, precision model, and "quadratic" precedent

• Median vs mean: Briefly justify why medians are preferred (e.g., skew/outliers).

• Precision modeling (Fig. 5): Your LME uses a quadratic term centered on fixation, which implicitly constrains the minimum/maximum precision to occur at the fixation/meridian. Given the paper's focus on how precision varies relative to fovea and across hemifields, this constraint may mask dissociations between peak precision and peak FJE. Consider a more flexible model (e.g., mixed effects cubic splines/GAM over flash position), or at least an unconstrained quadratic (not forced to have its extremum at the meridian). Reporting data driven minima would let readers evaluate whether precision and FJE peaks dissociate.

• "Quadratic transformation analogous to Albright &Desimone (1987)": We only looked at that paper briefly, but we believe it models receptive field/visuotopy precision as a LINEAR function of eccentricity, which we think is usually the case (a linear relationship), it is not obvious that your specific quadratic transform is warranted by that work. Please provide a more direct rationale.

Action: (i) Clarify/justify median choice and align error bars; (ii) re fit precision with a flexible position term (or present a supplemental analysis); (iii) revise the Albright &Desimone analogy or provide a clearer mapping.

7) Terminated vs continuing motion and the extrapolation account

• Your framework emphasizes extrapolation modulated by adaptation. However, there is a rich literature on terminated conditions that should be more fully integrated. For example, Sundberg et al. (2006) report a neural position shift in macaque V4 that persists even when motion is terminated at the flash, whereas human perceptual shift can diminish-implying dissociation between V4 retinotopic shifts and perception. This is relevant to your interpretation and should perhaps be discussed explicitly.

• Your own Experiment 1/2 results clearly show robust FJE and resurgence patterns and model based adaptation effects; please align the Discussion to explain why expectation based accounts (e.g., Coffey et al., 2019) are insufficient for your paradigm, and pinpoint where an adaptation driven extrapolation account better fits your data (e.g., the hemifield specific decay and midline resurgence that expectation alone would not predict).

Action: Add a subsection contrasting terminated condition findings and expectation accounts with your adaptation extrapolation account, including Sundberg et al. (neural) vs human (perceptual) dissociation and why your midline effects favor adaptation based explanations.

8) Claims about MST integration and the final take home message

• There is extensive discussion of hemispheric integration, but we don't think that is highly relevant to this data. To explain the participant behavior, how far could a participant get if they were a fully split-brain patient? The discussion of integration fails to explain why integration is needed. One case in which it would be needed is if the object flashed shortly before crossing the vertical meridian and its location is perceived on the other side of the meridian. That might well be in this data, but we don't think it is ever specifically called out by the authors.

• Even if there is reason to discuss hemispheric integration, the MST claim is intriguing but currently inferential. Phrasings like "likely occurring in area MST" and "solidifies motion extrapolation as the key mechanism" are too assertive relative to the data. Please soften to "consistent with integration in MST" and acknowledge alternative frameworks (feedback/oscillatory binding, top down modulation which have also been invoked extensively in the literature to explain illusions such as the FLE).

• The smooth pursuit paragraph is compelling, but it suggests the limitation "may serve as a driver" (typo: "diver") of pursuit. Consider reframing as a testable prediction (e.g., predicting reduced midline resurgence during pursuit) rather than a firm conclusion.

Action: Temper causal language about MST and pursuit; make them predictions or hypotheses for future work.

9) Eyetracking methodology. It's unclear how fixations (and saccades) were screened before/after the flash, especially since participants could move their eyes during the response period. Provide exclusion criteria (e.g., {plus minus}1{degree sign} window within [−50, +50] ms of the flash), the proportion of excluded trials, and whether excluding trials with pre flash saccades changes the results. This seems important given the strong claims about hemifield specific processing at the vertical meridian.

10) Effect of color. Given the previous report of color dependent magnitude differences, at least provide a supplementary check that color doesn't interact with midline resurgence or pre midline decay (even if the main effect replicates prior work).

11) On line 377, the authors acknowledge a foveopetal/foveofugal confound for their main conclusion that adaptation is driving things. Unless the authors have a strong argument against that interpretation, they need to mention this in the abstract, as well as discussing its probability or improbability more.

Figures, Reporting &Clarity

1. Terminology

◦ Replace "horizontal meridian" with "vertical meridian" if actually referring to hemifield crossings (multiple instances: Fig. 2 legend, Results text, and figure legends).

2. Statistics table phrasing Fix "Note statistics from tables using in text excluded" (garbled). Suggest: "Statistics reported inline are omitted from this table" if accurate.

3. Reproducibility We recommend providing a minimal, runnable analysis script (e.g., in R or Python) for the LME and exponential fits (with seed and package versions), and a data dictionary for trial wise variables (position, direction, color, condition, fixation block, response), as well as the data itself.

Specific / Line Level Points

• Intro, coherence of Bayesian framing: Where you write "The Bayesian perspective provides a unifying framework ... including the flash lag effect, FJE," consider noting that some position shifts (e.g., random motion cases) are not fully explained by simple Bayesian extrapolation, motivating your adaptation modulated approach.

• Discussion:

◦ Where you propose "initial binding ... in MT" and "integration in MST," soften and position as hypotheses consistent with receptive field sizes and bilateral coverage, not definitive localization.

◦ Fix typo "diver" → "driver"; check "motion extrapolation signal.in" → "signal in".

Suggestions for Additional Analyses (recommended, not all mandatory)

1. Unconstrained precision model Re analyze precision with a spline (GAM) mixed effects term over flash position (per fixation), to estimate where precision is maximal/minimal without assuming it is at the meridian. Report the position of best precision (and CI) per condition and compare to the position of maximal FJE to test for a dissociation.

2. Robustness of resurgence Provide a within participant bootstrap estimate of (a) pre midline decay constant and (b) post midline peak resurgence(magnitude and location), with CIs. This will help adjudicate the "earlier vs later crossing" issue.

3. Terminated vs continuing We weren't clear on the justification for different adaptation models for terminated and continuing conditions (line 274), nor on the interpretation (why should they be different). For terminated trials, consider fitting a state space or piecewise model to see whether the shape (linear vs exponential) differs reliably from continuing trials, and discuss why adaptation signatures differ (or not) between them.

References to Add (suggested)

• Cai, R. H., &Schlag, J. (2001). Asynchronous feature binding and the flash lag illusion. VSS Abstract. (Priority citation for the FJI.) [compiled_c...ighlighted | Word]

• Cottier, T. V., et al. (2023, 2025) on low cross illusion correlations, arguing against a single common mechanism (supports tempering cross illusion extrapolation claims).

• Consider adding any recent binding/temporal error/feedback perspectives relevant to feature-motion integration to balance the extrapolation account.

Suggested Text Edits (examples)

• Meridian phrasing (Fig. 2 legend and throughout): "The blue lines are the fits for flashes before crossing the vertical meridian, and the green lines are the fits for flashes after crossing the vertical meridian."

• Resurgence direction (Results, Exp 2): Choose one and stick to it; e.g., "Resurgence magnitude was greater when the midline was crossed earlier in the motion sequence (shorter pre midline history), consistent with less adaptation at crossing." or the opposite-depending on your re check-then align all numbers and the Discussion accordingly.

• MST claim (Discussion): "These findings are consistent with the idea that MST, with its larger bilateral receptive fields, integrates hemifield specific signals from MT, but our data do not localize this process."

Minor/Typos

• "horizontal meridian" → "vertical meridian" in Results and figure legends.

• "diver of an important oculomotor behaviour" → "driver".

• "signal.in" → "signal in".

• Clean the Stats Table note ("tables using ...") for readability.

About the process of writing this review, myself and a research student independently wrote comments. We then used a university-internal, secure (so that the information wouldn't be used to train models) enterprise version of Microsoft Copilot to collate our two sets of comments, after which we went through and made many edits to the collated version.

We hope these comments are useful to the authors and editor.
