# Supplementary material for: Hemifield-Specific Motion Extrapolation Reveals Limits of Interhemispheric Integration
Source: eNeuro. 2026 Aug 7;13(8):ENEURO.0004-26.2026. doi: 10.1523/ENEURO.0004-26.2026 (PMC13471930; doi:10.1523/ENEURO.0004-26.2026)
Supplement: Supplementary file 1 [file eneuro-13-ENEURO.0004-26.2026-s002.docx]

**Extended Data Figure 2-1. Experiment 1 FJE Magnitude Linear Mixed Effects Model with Direction of Motion.** Parameter estimates and test statistics for the FJE magnitude linear mixed-effects model that includes the direction of motion. Abbreviations: Path Len, path length (dva); Term, flash-terminating condition; RW, rightward motion (other parameters use leftward motion); Post-Mid, indicator for post-midline flashes; SE, standard error; CI, confidence interval. Model fit statistics: AIC = 46820.07, log-likelihood = **-**23383.03. Likelihood-ratio comparison against a null model: χ²(24) = 5476.09, p < .001.

| **Term** | **Estimate ± SE** | **z** | **p** | Random Effects SE |
| --- | --- | --- | --- | --- |
| Post-Mid | 0.1336 +/- 0.1647 | 0.811 | 0.4173 | 0.1977 |
| Post-Mid:RW | 0.5157 +/- 0.2234 | 2.308 | 0.0210 | - |
| Intercept | 1.6390 +/- 0.2044 | 8.019 | 1.06e^-15^ | 0.8558 |
| Path Len | -0.1997 +/- 0.0421 | -4.738 | 2.15e^-06^ | 0.1465 |
| Path Len:Post-Mid | 0.0948 +/- 0.0350 | 2.708 | 0.0068 | - |
| Path Len:Post-Mid:RW | -0.1009 +/- 0.0495 | -2.038 | 0.0416 | - |
| Path Len:RW | 0.0430 +/- 0.0351 | 1.225 | 0.2204 | - |
| Path Len:term | 0.1013 +/- 0.0351 | 2.886 | 0.0039 | - |
| Path Len:term:Post-Mid | -0.1103 +/- 0.0495 | -2.227 | 0.0259 | - |
| Path Len:term:Post-Mid:RW | 0.0889 +/- 0.0700 | 1.270 | 0.2041 | - |
| Path Len:term:RW | -0.0054 +/- 0.0496 | -0.109 | 0.9132 | - |
| RW | 0.0172 +/- 0.1208 | 0.142 | 0.8866 | 0.4377 |
| term | -1.2317 +/- 0.0648 | -19.013 | 1.32e^-80^ | - |
| term:Post-Mid | 0.2167 +/- 0.2235 | 0.970 | 0.3323 | - |
| term:Post-Mid:RW | -0.4880 +/- 0.3159 | -1.545 | 0.1224 | - |
| term:RW | -0.0939 +/- 0.0915 | -1.026 | 0.3050 | - |
