# Supplementary material for: Hemifield-Specific Motion Extrapolation Reveals Limits of Interhemispheric Integration
Source: eNeuro. 2026 Aug 7;13(8):ENEURO.0004-26.2026. doi: 10.1523/ENEURO.0004-26.2026 (PMC13471930; doi:10.1523/ENEURO.0004-26.2026)
Supplement: Supplementary file 2 [file eneuro-13-ENEURO.0004-26.2026-s003.docx]

**Extended Data Figure 2-2. Experiment 1 FJE Magnitude Linear Mixed Effects Model with Colour of Flash.** Parameter estimates and test statistics for the FJE magnitude linear mixed-effects model. Abbreviations: Path Len, path length (dva); Term, flash-terminating condition; Col-G/Y/B, colour of flash with G=green, Y=yellow, B=blue (note that a red flash was used as the base for parameter estimates); Post-Mid, indicator for post-midline flashes; SE, standard error; CI, confidence interval. Model fit statistics: AIC = 47709.98, log-likelihood = -23800.99. Likelihood-ratio comparison against a null model: χ²(51) = 4640.18, p < .001.

| **Term** | **Estimate ± SE** | **z** | **p** | Random Effects SE |
| --- | --- | --- | --- | --- |
| Post-Mid | -0.0234 +/- 0.2443 | -0.096 | 0.9238 | 0.1868 |
| Post-Mid:Col-G | 0.4062 +/- 0.3231 | 1.257 | 0.2088 | - |
| Post-Mid:Col-Y | 0.7432 +/- 0.3212 | 2.314 | 0.0207 | - |
| Post-Mid:Col-B | 0.5199 +/- 0.3199 | 1.625 | 0.1041 | - |
| Intercept | 1.5368 +/- 0.1605 | 9.574 | 1.03e^-21^ | 0.7348 |
| Path Len | -0.1929 +/- 0.1288 | -1.497 | 0.1343 | 0.1428 |
| Path Len:Post-Mid | 0.1378 +/- 0.0503 | 2.740 | 0.0061 | - |
| Path Len:Post-Mid:Col-G | -0.0518 +/- 0.0716 | -0.725 | 0.4688 | - |
| Path Len:Post-Mid:Col-Y | -0.1939 +/- 0.0712 | -2.723 | 0.0065 | - |
| Path Len:Post-Mid:Col-B | -0.1271 +/- 0.0711 | -1.788 | 0.0739 | - |
| Path Len:Col-G | -0.0447 +/- 0.0507 | -0.882 | 0.3777 | - |
| Path Len:Col-Y | 0.0611 +/- 0.0505 | 1.209 | 0.2266 | - |
| Path Len:Col-B | 0.0409 +/- 0.0506 | 0.810 | 0.4181 | - |
| Path Len:term | 0.1085 +/- 0.0506 | 2.143 | 0.0321 | - |
| Path Len:term:Post-Mid | -0.1595 +/- 0.0712 | -2.241 | 0.0250 | - |
| Path Len:term:Post-Mid:Col-G | 0.0684 +/- 0.1009 | 0.678 | 0.4976 | - |
| Path Len:term:Post-Mid:Col-Y | 0.1679 +/- 0.1009 | 1.665 | 0.0959 | - |
| Path Len:term:Post-Mid:Col-B | 0.1381 +/- 0.1006 | 1.373 | 0.1697 | - |
| Path Len:term:Col-G | 0.0250 +/- 0.0715 | 0.349 | 0.7270 | - |
| Path Len:term:Col-Y | -0.0414 +/- 0.0715 | -0.578 | 0.5630 | - |
| Path Len:term:Col-B | -0.0204 +/- 0.0714 | -0.286 | 0.7750 | - |
| Col-G | 0.3406 +/- 0.1552 | 2.195 | 0.0282 | - |
| Col-Y | 0.1496 +/- 0.1542 | 0.970 | 0.3320 | - |
| Col-B | -0.0556 +/- 0.1557 | -0.357 | 0.7212 | - |
| term | -1.2188 +/- 0.0936 | -13.021 | 9.28e^-39^ | - |
| term:Post-Mid | 0.4701 +/- 0.3203 | 1.468 | 0.1422 | - |
| term:Post-Mid:Col-G | -0.5688 +/- 0.4554 | -1.249 | 0.2117 | - |
| term:Post-Mid:Col-Y | -0.7472 +/- 0.4551 | -1.642 | 0.1006 | - |
| term:Post-Mid:Col-B | -0.6958 +/- 0.4531 | -1.536 | 0.1246 | - |
| term:Col-G | -0.1974 +/- 0.1323 | -1.493 | 0.1355 | - |
| term:Col-Y | -0.1576 +/- 0.1319 | -1.195 | 0.2320 | - |
| term:Col-B | 0.1173 +/- 0.1320 | 0.889 | 0.3742 | - |
