# Supplementary material for: Hemifield-Specific Motion Extrapolation Reveals Limits of Interhemispheric Integration
Source: eNeuro. 2026 Aug 7;13(8):ENEURO.0004-26.2026. doi: 10.1523/ENEURO.0004-26.2026 (PMC13471930; doi:10.1523/ENEURO.0004-26.2026)
Supplement: Supplementary file 3 [file eneuro-13-ENEURO.0004-26.2026-s004.docx]

**Extended Data Figure 3-1. Experiment 1 FJE Precision Path Length Linear Mixed Effects Model.** Parameter estimates and test statistics for the FJE precision linear mixed-effects model. Abbreviations: Path Len, path length (dva); Term, flash-terminating condition; SE, standard error; CI, confidence interval. Model fit statistics: AIC = −1230.97, log-likelihood = 623.48. Likelihood-ratio comparison against a null model: χ²(3) = 49.44, p < .001.

| **Parameter** | **Estimate ± SE** | **z** | **p** | Random Effects SE |
| --- | --- | --- | --- | --- |
| Intercept | 0.4619 +/- 0.0259 | 17.825 | 4.51e^-71^ | 1.8412 |
| Path Len | -0.0028 +/- 0.0017 | -1.666 | 0.0957 | - |
| Path Len:Term | 0.0028 +/- 0.0024 | 1.162 | 0.2452 | - |
| Term | -0.1804 +/- 0.0168 | -10.725 | 7.74e^-27^ | 0.9627 |
