# Supplementary material for: Hemifield-Specific Motion Extrapolation Reveals Limits of Interhemispheric Integration
Source: eNeuro. 2026 Aug 7;13(8):ENEURO.0004-26.2026. doi: 10.1523/ENEURO.0004-26.2026 (PMC13471930; doi:10.1523/ENEURO.0004-26.2026)
Supplement: Supplementary file 4 [file eneuro-13-ENEURO.0004-26.2026-s005.docx]

**Extended Data Figure 4-1. Experiment 2 FJE Magnitude Linear Mixed Effects Model with Motion Direction.** Parameter estimates and test statistics for the FJE magnitude linear mixed-effects model. Abbreviations: Path Len, path length (dva); Term, flash-terminating condition; Fix Loc, fixation location within sequence (early or late; central fixation otherwise); RW, rightward motion (other parameters use leftward motion); Post-Mid, indicator for post-midline flashes; SE, standard error; CI, confidence interval. Model fit statistics: AIC = 25698.23, log-likelihood = -12772.12. Likelihood-ratio comparison against a null model: χ²(74) = 2668.04, p < .001.

| **Term** | **Estimate ± SE** | **z** | **p** | Random Effects SE |
| --- | --- | --- | --- | --- |
| Post-Mid | 0.1991 +/- 0.9426 | 0.211 | 0.8327 | 0.1572 |
| Post-Mid:RW | 1.1590 +/- 1.3147 | 0.882 | 0.3780 | - |
| Fix Loc (Late) | -2.0507 +/- 0.5241 | -3.913 | 9.14e^-05^ | - |
| Fix Loc (Late):Post-Mid | 19.8748 +/- 5.8258 | 3.412 | 6.46e^-04^ | - |
| Fix Loc (Late):Post-Mid:RW | -16.4786 +/- 7.9899 | -2.062 | 0.0392 | - |
| Fix Loc (Late):RW | 3.0786 +/- 0.7286 | 4.225 | 2.39e^-05^ | - |
| Fix Loc (Early) | 4.5162 +/- 1.8923 | 2.387 | 0.0170 | - |
| Fix Loc (Early):Post-Mid | -5.0387 +/- 2.1062 | -2.392 | 0.0167 | - |
| Fix Loc (Early):Post-Mid:RW | 3.1796 +/- 2.8578 | 1.113 | 0.2659 | - |
| Fix Loc (Early):RW | -4.8499 +/- 2.5433 | -1.907 | 0.0565 | - |
| Intercept | 4.2706 +/- 0.5132 | 8.321 | 8.72e^-17^ | 0.7479 |
| Path Len | -0.2463 +/- 0.0607 | -4.061 | 4.89e^-05^ | 0.0529 |
| Path Len:Post-Mid | 0.0698 +/- 0.0797 | 0.876 | 0.3811 | - |
| Path Len:Post-Mid:RW | -0.0417 +/- 0.1112 | -0.375 | 0.7076 | - |
| Path Len:Fix Loc (Late) | 0.2049 +/- 0.0653 | 3.140 | 0.0017 | - |
| Path Len:Fix Loc (Late):Post-Mid | -1.2282 +/- 0.3436 | -3.575 | 3.51e^-04^ | - |
| Path Len:Fix Loc (Late):Post-Mid:RW | 0.9828 +/- 0.4710 | 2.087 | 0.0369 | - |
| Path Len:Fix Loc (Late):RW | -0.2096 +/- 0.0911 | -2.302 | 0.0213 | - |
| Path Len:Fix Loc (Early) | -0.7499 +/- 0.3636 | -2.062 | 0.0392 | - |
| Path Len:Fix Loc (Early):Post-Mid | 0.7628 +/- 0.3690 | 2.067 | 0.0387 | - |
| Path Len:Fix Loc (Early):Post-Mid:RW | -0.5657 +/- 0.4991 | -1.133 | 0.2571 | - |
| Path Len:Fix Loc (Early):RW | 0.7188 +/- 0.4913 | 1.463 | 0.1434 | - |
| Path Len:RW | -0.0239 +/- 0.0803 | -0.297 | 0.7664 | - |
| Path Len:term | 0.1514 +/- 0.0801 | 1.891 | 0.0587 | - |
| Path Len:term:Post-Mid | -0.0895 +/- 0.1120 | -0.799 | 0.4245 | - |
| Path Len:term:Post-Mid:RW | -0.0418 +/- 0.1570 | -0.266 | 0.7901 | - |
| Path Len:term:Fix Loc (Late) | -0.1764 +/- 0.0909 | -1.941 | 0.0522 | - |
| Path Len:term:Fix Loc (Late):Post-Mid | 1.1709 +/- 0.4845 | 2.417 | 0.0157 | - |
| Path Len:term:Fix Loc (Late):Post-Mid:RW | -0.7032 +/- 0.6737 | -1.044 | 0.2966 | - |
| Path Len:term:Fix Loc (Late):RW | 0.2295 +/- 0.1273 | 1.802 | 0.0715 | - |
| Path Len:term:Fix Loc (Early) | -0.0098 +/- 0.5012 | -0.020 | 0.9843 | - |
| Path Len:term:Fix Loc (Early):Post-Mid | -0.0020 +/- 0.5090 | -0.004 | 0.9969 | - |
| Path Len:term:Fix Loc (Early):Post-Mid:RW | -0.1192 +/- 0.6957 | -0.171 | 0.8640 | - |
| Path Len:term:Fix Loc (Early):RW | 0.0341 +/- 0.6844 | 0.050 | 0.9603 | - |
| Path Len:term:RW | 0.0947 +/- 0.1121 | 0.845 | 0.3983 | - |
| RW | -0.0177 +/- 0.6403 | -0.028 | 0.9780 | 0.5719 |
| term | -2.9280 +/- 0.6362 | -4.602 | 4.18e^-06^ | 0.5561 |
| term:Post-Mid | 0.1895 +/- 1.3300 | 0.142 | 0.8867 | - |
| term:Post-Mid:RW | -0.2313 +/- 1.8664 | -0.124 | 0.9014 | - |
| term:Fix Loc (Late) | 1.2372 +/- 0.7250 | 1.706 | 0.0879 | - |
| term:Fix Loc (Late):Post-Mid | -18.7081 +/- 8.2185 | -2.276 | 0.0228 | - |
| term:Fix Loc (Late):Post-Mid:RW | 11.5796 +/- 11.4287 | 1.013 | 0.3110 | - |
| term:Fix Loc (Late):RW | -2.5271 +/- 1.0157 | -2.488 | 0.0128 | - |
| term:Fix Loc (Early) | -0.8684 +/- 2.5895 | -0.335 | 0.7374 | - |
| term:Fix Loc (Early):Post-Mid | 1.8449 +/- 2.9056 | 0.635 | 0.5255 | - |
| term:Fix Loc (Early):Post-Mid:RW | -0.6750 +/- 3.9865 | -0.169 | 0.8655 | - |
| term:Fix Loc (Early):RW | 0.7278 +/- 3.5287 | 0.206 | 0.8366 | - |
| term:RW | -0.7748 +/- 0.8435 | -0.918 | 0.3583 | - |
