# Supplementary material for: Hemifield-Specific Motion Extrapolation Reveals Limits of Interhemispheric Integration
Source: eNeuro. 2026 Aug 7;13(8):ENEURO.0004-26.2026. doi: 10.1523/ENEURO.0004-26.2026 (PMC13471930; doi:10.1523/ENEURO.0004-26.2026)
Supplement: Supplementary file 5 [file eneuro-13-ENEURO.0004-26.2026-s006.docx]

**Extended Data Figure 5-1. Experiment 2 Bootstrap Peak Estimates Consistency Across Iterations.** The amount of iterations in which a local peak was identified for each condition of the experiment 2 bootstrap analysis procedure (see methods). The iteration counts are out of a total 5000 run for each condition. The * indicates conditions where the estimated 95% CI peak range covered no more than 3 actual flash locations.

| **VM Crossing Position** | **Flash-Continuing** | **Flash-Terminating** |
| --- | --- | --- |
| **Early** | 4935/5000 | 5000/5000 |
| **Middle** | *4996/5000 | 4955/5000 |
| **Late** | *4986/5000 | 4823/5000 |
