# Supplementary material for: Hemifield-Specific Motion Extrapolation Reveals Limits of Interhemispheric Integration
Source: eNeuro. 2026 Aug 7;13(8):ENEURO.0004-26.2026. doi: 10.1523/ENEURO.0004-26.2026 (PMC13471930; doi:10.1523/ENEURO.0004-26.2026)
Supplement: Supplementary file 6 [file eneuro-13-ENEURO.0004-26.2026-s007.docx]

**Extended Data Figure 6-1. Experiment 2 FJE Precision Path Length Linear Mixed Effects Model.** Parameter estimates and test statistics for the FJE precision linear mixed-effects model based on the length of the motion sequence. Abbreviations: Path Len, path length (dva); Term, flash-terminating condition; Fix Loc, fixation location within sequence (early or late; central fixation otherwise); SE, standard error; CI, confidence interval. Model fit statistics: AIC = 4731.15, log-likelihood = −2349.58. Likelihood-ratio comparison against a null model: χ²(11) = 144.33, p < .001.

| **Parameter** | **Estimate ± SE** | **z** | **p** | Random Effects SE |
| --- | --- | --- | --- | --- |
| Fix Loc (Late) | 0.8115 +/- 0.1895 | 4.282 | 1.85e^-05^ | - |
| Fix Loc (Early) | 0.8795 +/- 0.1895 | 4.641 | 3.47e^-06^ | - |
| Intercept | 1.6205 +/- 0.1768 | 9.165 | 4.97e^-20^ | 0.6825 |
| Path Len | 0.0183 +/- 0.0113 | 1.618 | 0.1056 | - |
| Path Len:Fix Loc (Late) | -0.0642 +/- 0.0160 | -4.021 | 5.79e^-05^ | - |
| Path Len:Fix Loc (Early) | -0.0374 +/- 0.0160 | -2.343 | 0.0191 | - |
| Path Len:Term | -0.0221 +/- 0.0160 | -1.384 | 0.1665 | - |
| Path Len:Term:Fix Loc (Late) | 0.0687 +/- 0.0226 | 3.042 | 0.0023 | - |
| Path Len:Term:Fix Loc (Early) | 0.0388 +/- 0.0226 | 1.719 | 0.0857 | - |
| Term | -0.5365 +/- 0.2096 | -2.559 | 0.0105 | 0.5306 |
| Term:Fix Loc (Late) | -0.7170 +/- 0.2680 | -2.676 | 0.0075 | - |
| Term:Fix Loc (Early) | -0.4833 +/- 0.2679 | -1.804 | 0.0712 | - |
